# Balancing Bulk Solution Chemistry and Cation Solvation for Stable Aqueous Aluminum Batteries

**DOI:** 10.1002/advs.76540

**Published:** 2026-07-09

**Authors:** Bei‐Er Jia, Gang Wu, Huoliang Gu, Jin Jie Liew, Dongping Chen, Jinxuan Song, Dan‐Yang Wang, Erhai Hu, Hong Han Choo, Yuzhu Liu, Yue Hu, Jinpeng Song, Anchun Tang, Zhenxiang Xing, Qiang Zhu, Chunshuang Yan, Chade Lv, Man‐Fai Ng, Qingyu Yan

**Affiliations:** ^1^ School of Materials Science and Engineering Nanyang Technological University Singapore Singapore; ^2^ Institute of High Performance Computing (IHPC), Agency for Science Technology and Research (A*STAR) Singapore Singapore; ^3^ MIIT Key Laboratory of Critical Materials Technology for New Energy Conversion and Storage, State Key Laboratory of Space Power‐Sources, School of Chemistry and Chemical Engineering Harbin Institute of Technology Harbin China; ^4^ Institute of Materials Research and Engineering (IMRE), Agency for Science Technology and Research (A*STAR) Singapore Singapore

**Keywords:** aqueous aluminum batteries, bulk solution, electrolyte design, hydrogen bond networks, solvation structure

## Abstract

The reversibility of aluminum (Al) metal anodes in aqueous batteries is limited by the high reactivity of bulk solution chemistry and the instability of strongly hydrated Al^3+^, yet electrolytes are typically optimized empirically without distinguishing these two effects. Here, we rationalize electrolyte design using a dual‐scale optimization framework that independently evaluates bulk solution effects and Al^3+^ solvation behavior through combined electrochemical measurements and molecular dynamics simulations. Trialkyl phosphate cosolvents are shown to disrupt the hydrogen bond network of water while partially replacing coordinated H_2_O in the Al^3+^ primary solvation shell, with distinct composition‐dependent responses at the bulk and solvation levels. An optimal electrolyte (40TMP) emerges from balancing suppressed water reactivity, moderate desolvation penalty, and fast ion transport, delivering an ionic conductivity of 24.6 mS cm^−1^ and stable Al||Al cycling for over 500 h. This work provides a rational framework for electrolyte optimization in aqueous aluminum electrochemistry.

## Graphical abstract

A dual‐scale electrolyte design balances bulk solution chemistry and cation solvation in aqueous aluminum batteries. Regulation of the bulk solution preserves high ionic conductivity and electrochemical stability, while modulation of the Al^3+^ solvation environment lowers desolvation penalties and suppresses hydrogen evolution. This balanced electrolyte enables stable and long‐life aqueous aluminum batteries.

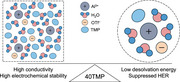

## 1 Introduction

Aqueous aluminum metal batteries (AAMBs) are promising candidates for post‐lithium energy storage owing to the ultrahigh theoretical capacities of aluminum (Al) metal (2980 mAh g^−1^, 8046 mAh cm^−3^) and its natural abundance in the Earth's crust (8.2 wt.%) [1, 2, 3, 4, 5, 6]. However, the practical application of Al metal anodes is severely hindered by their poor cycling stability. Parasitic reactions, including hydrogen evolution (HER), surface passivation, and corrosion, rapidly consume active Al and destabilize the electrode–electrolyte interface [7, 8, 9, 10, 11]. These failure pathways originate from the intrinsic aqueous environment. Al^3+^ ions are strongly hydrated, and the coordinated water molecules are more susceptible to electrochemical reduction than free water, triggering vigorous HER during charging [12]. Meanwhile, bulk water maintains high activity through its interconnected hydrogen bond (HB) network, further constraining the electrochemical stability window of aqueous electrolytes [13].

To address the intrinsic instability of aqueous aluminum electrochemistry, it is necessary to consider two coupled but physically distinct factors, namely the properties of the bulk solution environment and the Al^3+^ cation dynamics. From the bulk perspective, suppressing water activity by weakening the extended HB network is essential for mitigating HER and corrosion [14, 15], yet excessive disruption of the solvent matrix often compromises ionic conductivity, viscosity, and practical safety [13, 16]. From the cation perspective, Al^3+^ is strongly hydrated, and regulating its primary solvation structure is critical for modulating interfacial reactions associated with coordinated water and stabilizing Al stripping and plating processes [17, 18, 19, 20, 21, 22, 23, 24]. However, excessive modification of the solvation shell can introduce substantial desolvation penalties and slow interfacial kinetics [25, 26]. In practice, these bulk solution effects and cation dynamics respond differently to electrolyte composition and are frequently treated in an implicit or coupled manner. As a result, electrolyte formulations are commonly optimized through empirical electrochemical screening, without explicitly disentangling bulk solution effects from cation solvation behavior. This lack of separation obscures the mechanistic origin of optimal compositions and limits the transferability of electrolyte design principles across systems.

Here, we rationalize electrolyte design for aqueous aluminum electrochemistry by jointly considering bulk solution effects and Al^3+^ cation dynamics, and by independently evaluating their composition‐dependent responses. Four trialkyl phosphates were systematically investigated as model cosolvents. Among them, trimethyl phosphate (TMP) emerges as the optimal choice owing to its relatively high dielectric constant and appropriate coordination strength with Al^3+^. Spectroscopic analyses, electrochemical measurements, and molecular dynamics (MD) simulations reveal that TMP disrupts the HB network of water, lowers water activity, and partially replaces coordinated water within the Al^3+^ solvation shell, with distinct composition‐dependent responses at the bulk and solvation levels. The optimized formulation (40TMP) achieves a well‐balanced compromise among water reactivity suppression, moderate desolvation penalty, and high ionic conductivity (24.6 mS cm^−1^). This tailored solvation environment effectively suppresses HER, alleviates Al corrosion, and promotes the formation of a robust hybrid organic–inorganic solid–electrolyte interphase (SEI). As a result, the electrolyte delivers significantly improved electrochemical performance, including >500 h of stable Al||Al cycling at 0.1 mAh cm^−2^ and a sevenfold increase in full‐cell lifespan relative to the baseline electrolyte. Furthermore, integrating a tin interfacial layer (Sn@Al) further enhances anode reversibility, enabling the Sn@Al|40TMP|polyaniline full cell to retain 70% of its capacity after 400 cycles. This dual‐scale framework links bulk solution chemistry, cation solvation, and interfacial SEI formation, providing a rational basis for electrolyte optimization in aqueous aluminum batteries.

## 2 Results and Discussion

### 2.1 Rational Screening of Trialkyl Phosphate Cosolvents

**FIGURE 1:**
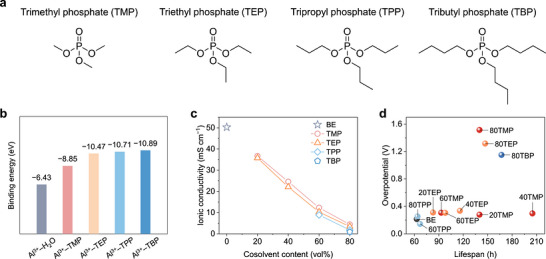
(a) Molecular structures of TMP, TEP, TPP, and TBP. (b) Calculated binding energies of Al^3+^ with the four phosphate molecules and H_2_O. (c) Ionic conductivities of electrolytes containing different trialkyl phosphate cosolvents at various volume fractions. (d) Galvanostatic cycling performance of Al||Al symmetric cells with twelve electrolytes (0.1 mA cm^−2^, 0.1 mAh cm^−2^).

Based on previous studies on aqueous zinc‐ion batteries, we list the key physicochemical parameters of representative candidates in Table S1, including melting and boiling points, flash point, viscosity, donor number, dielectric constant, flammability, and water miscibility. Among these, trimethyl phosphate (TMP) and triethyl phosphate (TEP) stood out because of their broad liquid‐phase stability windows and inherent non‐flammability, both of which contribute to enhanced electrolyte safety. Since TMP and TEP belong to the trialkyl phosphate family, two additional commercially available analogues, tripropyl phosphate (TPP) and tributyl phosphate (TBP), were also examined. These organophosphorus compounds act as flame‐retardant molecules that contain three alkoxy substituents attached to a central phosphoryl (P═O) group (Figure 1a). The P═O moiety serves as a strong HB acceptor, enabling these molecules to interact with water and potentially alter its HB structure. Density functional theory (DFT) calculations were carried out to determine the binding energies between Al^3+^ and each of the four phosphate molecules, as well as with water (Figure 1b). The results show that Al^3+^ interacts more strongly with all four phosphates than with water, and that the binding energy increases sublinearly with the elongation of the alkoxy chain. This observation suggests that all members of the trialkyl phosphate family exhibit strong coordination with Al^3+^ and possess considerable potential to regulate the solvation structure of Al^3+^ ions in aqueous electrolytes.

Subsequently, a series of electrolytes employing these four molecules as cosolvents was prepared, with 1 M Al(OTf)_3_ serving as the baseline electrolyte (BE). Electrolytes containing *x* vol% TMP were denoted as *x*TMP (*x* = 20, 40, 60, 80), and the same notation was applied to TEP, TPP, and TBP systems. As shown in Figure S1, electrolytes with TMP and TEP remained clear and homogeneous across the entire compositional range. In contrast, those containing TPP and TBP exhibited phase separation at low cosolvent contents and became uniform only at higher volume fractions (60TPP, 80TPP, and 80TBP). Consequently, twelve formulations were included in the subsequent analysis. From the perspective of bulk solution effects, ionic conductivity serves as a sensitive indicator of the trade‐off between suppressed water activity and preserved ion transport. As displayed in Figure 1c, the BE exhibited an ionic conductivity of 50.3 mS cm^−1^. At identical volume fractions, the conductivity followed the trend TMP > TEP > TPP > TBP, which correlates well with their dielectric constants (TMP, 21.3; TEP, 13.0; TPP, 10.9; TBP, 8.3). Solvents with higher dielectric constants can more effectively dissociate cations and anions, thereby facilitating ion transport [27]. For each phosphate series, the ionic conductivity decreased with increasing cosolvent content. This behavior arises from two concurrent factors. First, all trialkyl phosphates possess lower dielectric constants than water (78.4), and thus a higher cosolvent fraction reduces the overall dielectric constant of the electrolyte. Second, their viscosities are greater than that of water. According to the Nernst–Einstein relation, ionic conductivity is proportional to the diffusion coefficient. Meanwhile, the Stokes–Einstein relation $D=\frac{k_{B}T}{6\pi \eta r}$ indicates that diffusion coefficient *D* is inversely proportional to viscosity η, where *k_B_* is Boltzmann constant, *T* is temperature, and *r* is solvation radius. Consequently, higher viscosity generally suppresses ionic conductivity.

Galvanostatic cycling tests were then conducted on Al||Al symmetric cells using the twelve electrolytes at 0.1 mA cm^−2^ and a capacity of 0.1 mAh cm^−2^ to evaluate the influence of the cosolvents (Figure 1d and Figure S2). In the BE, the cell exhibited a relatively low overpotential, yet its lifespan was limited to less than 70 h because of severe hydrogen evolution and corrosion. In comparison, the cells employing 80TMP, 80TEP, or 80TBP maintained operation for more than 140 h, but the overpotentials exceeded 1.1 V, indicating pronounced polarization and sluggish interfacial kinetics. Among all tested systems, the cell containing 40TMP delivered the most balanced performance, combining a moderate overpotential below 0.3 V with an extended cycling stability exceeding 200 h. Notably, this optimal concentration does not correspond to the maximum suppression of water reactivity or the highest ionic conductivity, but instead reflects a balance between competing bulk solution constraints. The remarkable performance of 40TMP prompted us to focus subsequent investigations on the TMP‐based electrolytes.

### 2.2 Bulk Solution Chemistry and Electrochemical Stability

**FIGURE 2:**
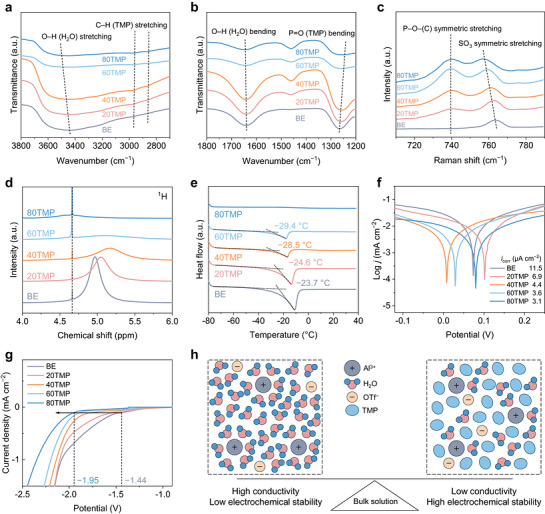
(a) FTIR spectra of electrolytes with varying TMP content, showing shifts in O─H (H_2_O) stretching and C─H (TMP) stretching modes. (b) FTIR spectra highlighting changes in O─H (H_2_O) bending and P═O (TMP) bending vibrations. (c) Raman spectra showing the evolution of P─O─C symmetric stretching and SO_3_ symmetric stretching bands with increasing TMP content. (d) ^1^H NMR spectra of electrolytes. (e) DSC curves of BE and 40TMP electrolytes. (f) Linear polarization curves of Al electrodes in symmetric cells at 0.5 mV s^−1^. (g) LSV of electrolytes at 5 mV s^−1^. (h) Schematic illustration of the trade‐off between ionic conductivity and electrochemical stability in the bulk solution phase.

FTIR spectra were collected to investigate the evolution of HB interactions in the electrolytes with increasing TMP content (Figure 2a,b). The C─H and P═O vibrational bands of TMP gradually shift to lower wavenumbers, suggesting that TMP engages in stronger intermolecular interactions, likely via HB with water molecules [28]. In contrast, both the O─H stretching (3600–3400 cm^−1^) and bending (∼1640 cm^−1^) modes of water shift to higher wavenumbers as TMP content increases. This blue shift of the O─H vibrational modes indicates that the native H_2_O─H_2_O HB network is weakened as TMP competes for hydrogen bonding with water [29]. Because the phosphoryl oxygen in TMP possesses higher electron density and stronger HB accepting capability than water, TMP preferentially forms P═O···H─O interactions that disrupt the cooperative H_2_O─H_2_O HB network. The loss of this cooperative network shortens and strengthens the intrinsic O─H covalent bond, as reflected by the blue‐shifted O─H stretching band. In addition, the strong and directional TMP–H_2_O interaction restricts proton mobility and further stabilizes water molecules, making it less prone to HER [30]. Raman spectroscopy was further used to probe vibrational features (Figure 2c). The band at 740 cm^−1^ is attributed to the symmetric stretching of the P─O─(C) group in TMP, while the SO_3_ symmetric stretching band of OTf^−^ shows a gradual red shift, reflecting changes in its chemical environment. The ^1^H nuclear magnetic resonance (NMR) spectra (Figure 2d) display a distinct two‐stage evolution of the water resonance upon increasing TMP content. From BE to 40TMP, the water peak progressively shifts downfield (from 4.97 to 5.17 ppm), indicative of enhanced deshielding caused by the formation of strong P═O···H─O HBs between TMP and water [31]. At higher TMP contents (60TMP and 80TMP), the resonance moves upfield and becomes markedly broadened, reflecting the collapse of the native water HB network [32].

Differential scanning calorimetry (DSC) results (Figure 2e) further support this conclusion. 60TMP exhibits a freezing point that is 5.7°C lower than that of the BE, while 80TMP shows no detectable endothermic peak. The observed freezing point depression can be attributed to the disruption of the HB network among water molecules [33]. To further assess interfacial stability, linear polarization measurements were carried out on Al electrodes (Figure 2f). The corrosion current density (*i*_corr_) decreases markedly with increasing TMP content, from 11.5 µA cm^−2^ in the BE to 6.9, 4.4, 3.6, and 3.1 µA cm^−2^ for 20TMP, 40TMP, 60TMP, and 80TMP, respectively, indicating that TMP effectively suppresses Al corrosion. The cathodic linear sweep voltammetry (LSV) curves clearly show that the HER onset potential shifts negatively with increasing TMP content (Figure 2g). Using 0.1 mA cm^−2^ as the threshold, the HER onset in 80TMP is 0.51 V more negative than that in BE. This delayed HER activation reflects a decreased water reactivity. Long‐term chemical stability was further evaluated by soaking Al electrodes in BE and 40TMP for up to seven days (Figure S3). In BE, the (111) Al diffraction peak exhibits a pronounced decay, indicating progressive corrosion, whereas its intensity remains essentially unchanged in 40TMP, confirming substantially reduced surface degradation. Figure 2h illustrates the macroscopic trade‐off introduced by cosolvent addition. Increasing TMP content disrupts the HB network and enhances the electrochemical stability of water, as reflected by the depressed freezing point, reduced corrosion current, and delayed HER onset. However, this stabilization is accompanied by a loss of ionic conductivity arising from decreased solvent dielectric constant and increased viscosity (Figure 1c). Consequently, 40TMP emerges as the optimal composition, as corroborated by the symmetric cell performance in Figure 1d. In addition, Al||Cu asymmetric cell measurements across different TMP contents show that 40TMP delivers the highest average Coulombic efficiency (CE) (Figure S4), further supporting its selection as the optimal composition. The ionic conductivity of 40TMP is 24.6 mS cm^−1^, nearly half that of the BE (Figure S5), yet still substantially higher than values reported for other aqueous Al(OTf)_3_‐based electrolytes containing organic cosolvents or additives (Table S2). This demonstrates that 40TMP achieves the desired balance between suppressing water reactivity and sustaining efficient ion transport. In addition to electrochemical stability, the thermal safety of the electrolyte was also evaluated. Combustion tests (Figure S6) show that pristine and BE‐wetted separators ignite rapidly and burn completely, whereas the 40TMP‐wetted separator remains structurally intact after 30 s, with only surface darkening. This flame retardancy stems from the radical‐trapping behavior of TMP in the gas phase, which interrupts chain branching and suppresses flame propagation [34].

### 2.3 Al^3+^ Solvation Shell Reconstruction

**FIGURE 3:**
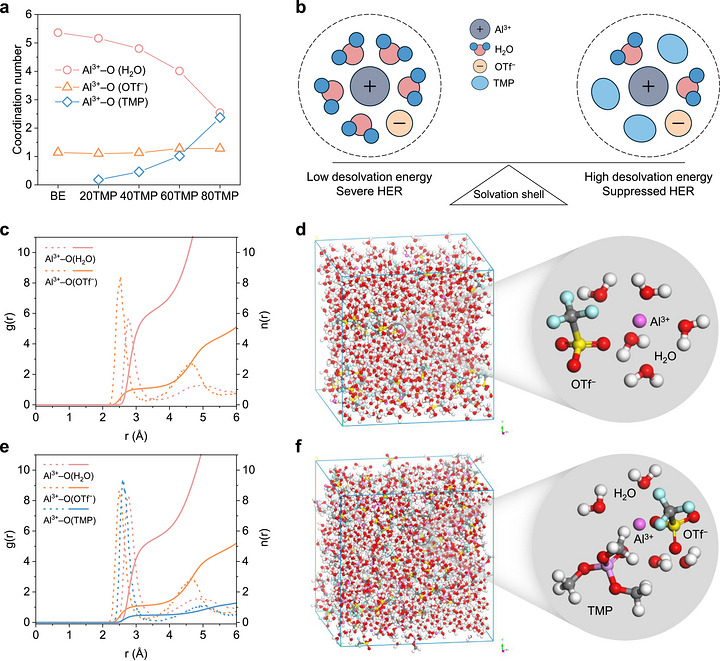
(a) Average coordination numbers of Al^3+^ obtained from MD simulations at different TMP contents. (b) Schematic illustration of the trade‐off between Al^3+^ desolvation energy and interfacial HER activity at the solvation shell level. Radial distribution function g(r) and coordination number n(r) of Al^3+^ in (c) BE and (e) 40TMP electrolytes obtained from MD simulations. Representative snapshots of simulation boxes and enlarged views of the primary solvation shells of Al^3+^ in (d) BE and (f) 40TMP electrolytes.

Beyond bulk solution effects, the interfacial behavior of Al^3+^ is governed by its primary solvation structure and desolvation dynamics. To elucidate the evolution of the Al^3+^ solvation environment upon introducing TMP, MD simulations were performed. The number of ions and molecules used for constructing the simulation cells is summarized in Table S3. The close agreement between the simulated and experimentally measured densities validates the reliability of the simulation setup. For each electrolyte composition, four configurations were simulated, and the resulting Al^3+^ coordination numbers (CNs) are compiled in Table S4 and plotted in Figure 3a. The average Al^3+^–O(OTf^−^) coordination remains nearly constant (1.1–1.3) across all compositions, indicating that TMP addition primarily affects the replacement of coordinated water rather than anion. The evolution of the local Al^3+^ coordination environment was further probed by ^27^Al NMR spectroscopy (Figure S7). Compared with the BE, the dominant ^27^Al resonance progressively shifts, broadens, and decreases in intensity with increasing TMP content, indicating gradual perturbation of the local Al^3+^ chemical environment upon TMP addition. At higher TMP contents, the spectral evolution becomes more pronounced, with the emergence of an additional broad resonance at a lower chemical shift, suggesting increasingly heterogeneous Al^3+^ coordination environments. These spectral changes indicate progressive modification of the local Al^3+^ coordination environment upon TMP addition, in agreement with the composition‐dependent solvation evolution predicted by MD simulations. Detailed analysis of the MD‐derived radial distribution functions provides further insight into the structural origin of this evolution. In BE, Al^3+^ is predominantly hydrated, as evidenced by the first Al^3+^–O(H_2_O) peak at 2.8 Å and a high CN of 5.42 (Figure 3c), indicating a water‐dominated primary solvation shell. In contrast, the Al^3+^–O(H_2_O) coordination decreases to 4.82 in 40TMP (Figure 3e), accompanied by the emergence of a new peak at 2.6 Å corresponding to Al^3+^–O(TMP) interactions with a CN of 0.47, indicating the incorporation of TMP molecules into the inner solvation shell. Representative snapshots of simulation cells and Al^3+^ solvation environments in BE and 40TMP (Figure 3d,f) clearly show that in 40TMP, one TMP molecule replaces a water molecule in the primary solvation shell. Compared with free water, coordinated water is more susceptible to reduction because the strong Lewis acidity of Al^3+^ polarizes and weakens the O─H bonds, making solvated water more readily reduced at the electrode interface [12, 35]. Reducing the number of coordinated water molecules helps mitigate HER at the Al–electrolyte interface during charging [36], which is crucial because HER directly competes with Al plating. However, excessive replacement of water by TMP is undesirable. For instance, in 80TMP, an average of 2.37 TMP molecules coordinate with Al^3+^. Thus, while decreasing coordinated water suppresses interfacial HER (Figure 2g), the strong Al^3+^–TMP binding (Figure 1b) simultaneously increases the desolvation barrier of Al^3+^, deteriorating deposition kinetics. Figure 3b schematically illustrates this solvation‐level trade‐off between Al^3+^ desolvation energy and interfacial HER activity. As TMP partially replaces coordinated water, the desolvation energy of Al^3+^ increases, making it more difficult for Al^3+^ to shed its solvation shell during interfacial charge transfer processes. Striking a balance between these competing effects, 40TMP achieves a favorable solvation structure, effectively suppressing HER while maintaining reasonable desolvation kinetics.

### 2.4 Solid Electrolyte Interphase Investigation

**FIGURE 4:**
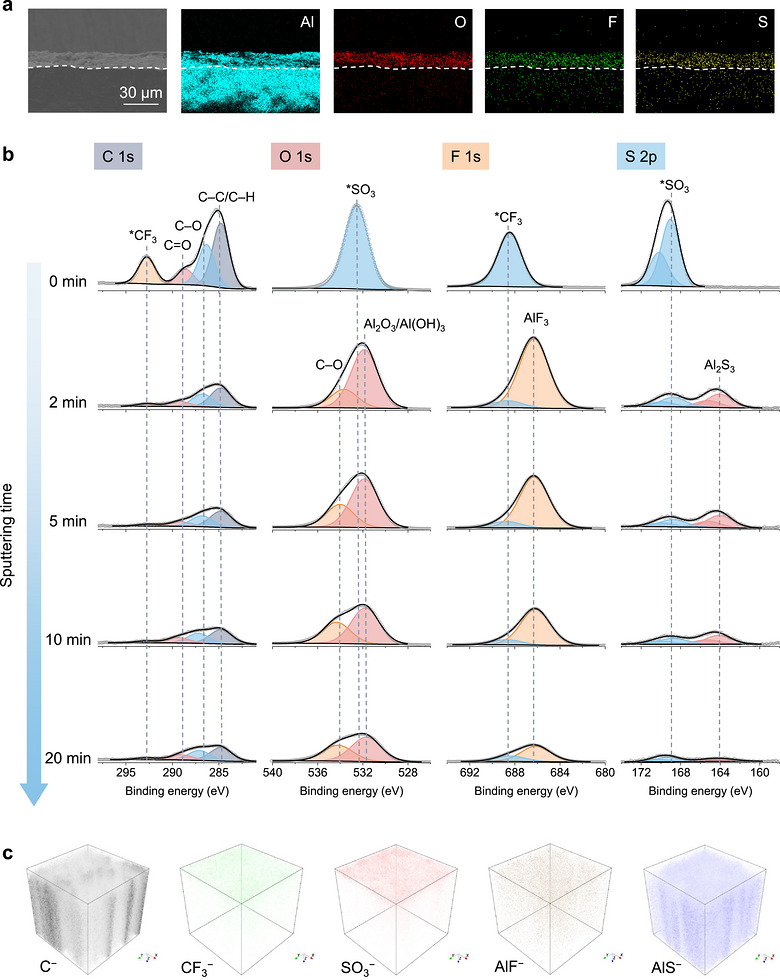
(a) Cross‐sectional SEM image and corresponding EDS elemental maps of the Al electrode after cycling in an Al|40TMP|Al symmetric cell (0.05 mA cm^−2^, 0.1 mAh cm^−2^, 25 cycles). (b) High‐resolution C 1s, O 1s, F 1s, and S 2p XPS spectra of the Al electrode cycled in 40TMP, collected after 0, 2, 5, 10, and 20 min of Ar^+^ sputtering. (c) ToF‐SIMS 3D elemental distributions of representative SEI fragments on the Al electrode cycled in 40TMP.

Recent studies have shown that cosolvents can induce the formation of SEI even in dilute aqueous battery systems, opening new opportunities for interfacial engineering [37, 38]. To investigate the surface evolution of the Al electrode, we conducted detailed multiscale characterization on Al foils retrieved from Al|40TMP|Al symmetric cells (0.05 mA cm^−2^, 0.1 mAh cm^−2^, 25 cycles). Cross‐sectional scanning electron microscopy (SEM) combined with energy‐dispersive X‐ray spectroscopy (EDS) elemental mapping reveals a distinct interfacial layer covering the Al surface. This layer is enriched in oxygen (O), fluorine (F), and sulfur (S) (Figure 4a), indicating the in situ formation of an interphase during cycling. Notably, no phosphorus signal is detected. This result indicates that TMP primarily acts as a solvation and bulk environment regulator rather than a direct SEI‐forming species. To further resolve the chemical composition and depth distribution of this interphase, high‐resolution X‐ray photoelectron spectroscopy (XPS) spectra were collected at increasing Ar^+^ sputtering times (Figure 4b). Before sputtering, the C 1s region displays prominent CF_3_ species at 292.8 eV along with other organic moieties originating from adsorbed OTf^−^ and its decomposition products [39]. As sputtering proceeds, these organic features gradually diminish. In the O 1s region, the initial SO_3_‐related signal vanishes after the first sputtering, accompanied by the emergence of dominant C─O (534.1 eV) and alumina (531.9 eV) signals. The F 1s spectra evolve from a single CF_3_ component at 688.5 eV to a dominant AlF_3_ contribution at 686.3 eV after sputtering. The S 2p spectra show a similar progression. Surface signals correspond to SO_3_ species near 169.0 eV, while sputtering reveals reduced sulfur species (Al_2_S_3_) near 164.0 eV [40]. These results collectively indicate that cycling leads to the formation of a chemically graded SEI with an organic‐rich outer region and an inorganic‐rich inner region composed mainly of AlF_3_ and Al_2_S_3_. These inorganic phases arise from the decomposition of OTf^−^ and its interfacial reactions with Al metal.

Complementary time‐of‐flight secondary ion mass spectrometry (ToF‐SIMS) 3D reconstructions provide additional spatial insight into the distribution of characteristic fragments, including C^−^, CF_3_^−^, SO_3_^−^, AlF^−^, and AlS^−^ (Figure 4c). Organic fragments (CF_3_^−^ and SO_3_^−^) are concentrated in the outer region of the interphase, whereas inorganic fragments (AlF^−^ and AlS^−^) are enriched in the inner region. Together, these analyses reveal that cycling in the 40TMP electrolyte produces a hybrid interphase architecture. The outer, organic‐rich region provides mechanical compliance, accommodates volume changes during Al stripping and plating, and partially shields the Al surface from direct contact with water. The inner, inorganic‐rich region composed of Al salts supports Al^3+^ transport while protecting the Al metal from water‐driven parasitic reactions [41, 42]. Such a configuration is expected to benefit both kinetic reversibility and long‐term interfacial stability.

For comparison, the Al electrode cycled in BE was also analyzed. SEM‐EDS (Figure S8) and XPS (Figure S9) reveal similar elemental types and chemical components, which are consistent with the MD results showing that the number of OTf^−^ anions in the Al^3+^ solvation shell remains essentially unchanged between BE and 40TMP. Since OTf^−^ is the primary precursor to the inorganic SEI, the fundamental chemistry is similar in both systems. However, ToF‐SIMS (Figure S10) shows dramatically weaker fragment signals in the BE case, indicating that the interphase formed in BE is much less substantial. This can be attributed to the dominance of water in the solvation shell, which promotes HER during charging. The resulting gas evolution can physically disrupt or remove nascent interphase species, thereby preventing the growth of a stable SEI [43]. In contrast, in the 40TMP electrolyte, TMP replaces part of the coordinated water in the solvation shell, which mitigates HER. Although TMP itself hardly contributes to the chemical composition of the SEI, it indirectly facilitates the development of a more robust and continuous SEI by suppressing interfacial HER.

### 2.5 Electrochemical Validation of the Balanced Solvent Composition

**FIGURE 5:**
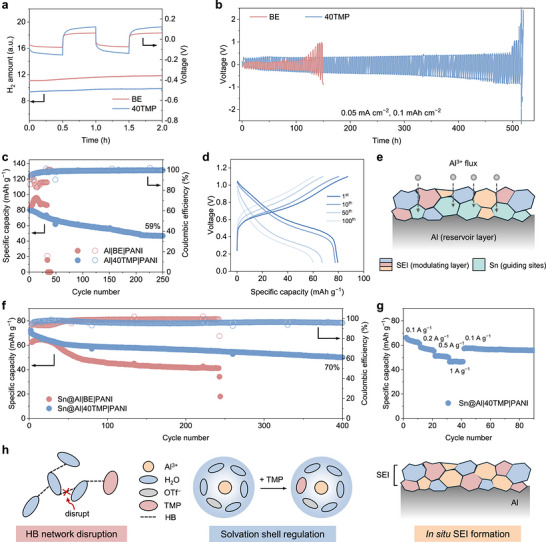
(a) DEMS measurements of Al||Al symmetric cells with BE and 40TMP electrolytes, conducted at 0.05 mA cm^−2^ with alternating 0.5 h discharge/charge steps. (b) Galvanostatic cycling of A||Al symmetric cells with BE and 40TMP electrolytes at 0.05 mA cm^−2^ and 0.1 mAh cm^−2^. (c) Cycling performance of Al||PANI full cells using BE and 40TMP electrolytes at 0.1 A g^−1^. (d) Voltage profiles of the Al|40TMP|PANI full cell at selected cycles. (e) Schematic illustration of the interfacial structure of the Sn@Al electrode during charging. (f) Cycling performance of Sn@Al||PANI full cells with BE and 40TMP electrolytes at 0.1 A g^−1^. (g) Rate performance of the Sn@Al|40TMP|PANI full cell under varying current densities. (h) Schematic illustration of TMP‐induced bulk and solvation shell restructuring, along with the formation of a robust SEI, leading to improved AAMB performance.

Differential electrochemical mass spectrometry (DEMS) further verifies the effectiveness of TMP in mitigating side reactions: the 40TMP electrolyte generates significantly less H_2_ during Al stripping/plating (Figure 5a). This improvement directly translates into electrochemical performance. The Al||Al symmetric cell with 40TMP sustains stable cycling for over 500 h at 0.05 mA cm^−2^ and 0.1 mAh cm^−2^, whereas the BE‐based cell fails within 120 h (Figure 5b). Consistently, asymmetric cell measurements show that, after stabilization over 400–500 cycles, the CE in 40TMP is 15.8% higher than that in BE (Figure S11), further supporting reduced parasitic reactions and improved Al utilization during Al plating/stripping. Having established superior interfacial stability enabled by 40TMP, identifying a cathode compatible with this electrolyte became essential for full‐cell construction [44]. We initially evaluated our previously reported manganese‐based Al*_x_* MnO_2_, known for its high‐voltage plateau and substantial capacity [45]. However, as shown in Figure S12, the Al|40TMP|Al*_x_* MnO_2_ cell delivers much lower capacity than its BE counterpart. This behavior further reflects the reduced proton activity in 40TMP, which is a direct consequence of bulk water regulation. Mn‐based cathodes typically rely on proton‐coupled intercalation [46] or dissolution–redeposition processes [47, 48], both of which diminish under proton‐deficient conditions. Therefore, a cathode that operates independently of proton chemistry is required [49, 50]. Recent studies have shown that polyaniline (PANI) stores charge primarily through reversible nitrogen‐centered redox coupled with the coordinated insertion of Al^3+^ and OTf^−^ ions in aqueous Al(OTf)_3_ electrolytes [17], making it a suitable candidate for the 40TMP system. PANI was synthesized following established procedures [51] and confirmed by structural characterization (Figure S13), whose XRD pattern exhibits three characteristic peaks corresponding to the (011), (020), and (200) reflections. The Al||PANI cells with BE and 40TMP display nearly identical cyclic voltammetry (CV) profiles (Figure S14) and voltage curves (Figure S15), consistent with previous reports [52], indicating identical redox chemistry in both electrolytes. As shown in Figure 5c, both Al|BE|PANI and Al|40TMP|PANI deliver similar initial capacities (∼80 mAh g^−1^). However, the Al|BE|PANI cell exhibits poor CE (87.5% on average) and collapses within only 33 cycles due to severe parasitic reactions, including HER and corrosion, which rapidly thin the Al electrode and ultimately cause loss of electrical contact. In contrast, Al|40TMP|PANI maintains an average CE of 99.0% and retains 59% of its initial capacity after 250 cycles, owing to the reduced side reactions. Post‐cycling SEM images of Al electrodes from full cells show that the Al surface in 40TMP remains relatively more intact than that in BE, which exhibits more severe surface degradation features (Figure S16). Nevertheless, its capacity retention remains suboptimal. The widening gap between the charge and discharge plateaus (Figure 5d) reveals increasing polarization arising from the accumulation of passivation layers on the Al surface, which hinder both charge transfer and Al^3+^ transport.

To further improve full‐cell performance, we introduced a proof‐of‐concept surface modification to the Al anode [53]. Tin (Sn) has been identified as an effective substrate for underpotential deposition (UPD) of Al and possesses a high HER overpotential that helps suppress hydrogen evolution [54, 55]. An Sn interlayer was formed on Al via a facile displacement reaction in aqueous SnCl_4_ solution (Figure S17), denoted Sn@Al. Longer immersion times produce greater Sn coverage and larger grains (Figure S18). An immersion duration of 45 min was selected as optimal, considering the trade‐off between insufficient Sn coverage, which offers limited benefits, and excessive Sn, which may hinder Al^3+^ release from the underlying substrate. As schematically illustrated in Figure 5e, the modified electrode consists of three functional components: the Al substrate as the Al^3+^ reservoir, the Sn layer serving as UPD‐active nucleation sites, and the in situ‐formed SEI during cycling, regulating Al^3+^ flux, mitigating parasitic reactions, and partially protecting Al from oxidation. With this design, the Sn@Al|BE|PANI cell achieves 240 cycles, far outperforming the unmodified Al|BE|PANI cell (Figure 5f). Combining the Sn interlayer with the 40TMP electrolyte yields robust performance: the Sn@Al|40TMP|PANI full cell retains 70% of its initial capacity after 400 cycles and shows excellent rate capability, maintaining >70% of its capacity at 1 A g^−1^ relative to 0.1 A g^−1^ (Figure 5g), attributable to enhanced interfacial transport kinetics. To further understand the origin of the enhanced cycling stability, post‐cycling cross‐sectional SEM and elemental mapping analyses were performed on the cycled Sn@Al electrode (Figure S19). A relatively continuous interfacial region is observed after prolonged cycling, in which Sn remains distributed together with electrolyte‐derived F‐ and S‐containing species near the electrode surface, indicating the formation of an electrolyte‐derived interphase. Sputtering‐assisted XPS measurements further reveal clear compositional evolution across the interphase (Figure S20). Before sputtering, the outermost surface is dominated by organic electrolyte‐derived species, including CF_3_ and SO_3_‐containing components associated with OTf^−^ residues/decomposition products. After sputtering, inorganic‐rich interphase species become more prominent, including AlF_3_/SnF*_x_*‐like fluorides, reduced sulfur species, SnO*_x_*, and Sn° components. These results suggest that the Sn interlayer not only serves as a favorable nucleation substrate for Al deposition but also participates in the formation of a chemically evolved interphase during cycling. Such an interphase is expected to regulate interfacial Al^3+^ transport while partially mitigating parasitic reactions and HER‐related interfacial degradation, thereby contributing to the improved long‐term cycling stability of the Sn@Al‐based full cells.

Practical considerations further highlight the advantages of 40TMP. Compared with previously reported additives or cosolvents for AAMBs, the 40TMP formulation incurs only a 7% cost increase relative to BE (Figure S21 and Table S5), demonstrating the economic viability of TMP‐based electrolyte engineering. Figure 5h summarizes the mechanism by which TMP enables stable Al cycling. At the bulk level, TMP disrupts the HB network and lowers water activity, thereby suppressing HER and corrosion that would otherwise destabilize the Al surface. At the solvation shell level, TMP partially replaces H_2_O in the primary solvation shell of Al^3+^, reducing the amount of coordinated water that is prone to interfacial reduction while avoiding the excessive desolvation penalty associated with high TMP contents. These bulk and solvation shell effects cooperate to promote the formation of a robust, inorganic‐rich SEI that blocks parasitic reactions and stabilizes Al deposition. The 40TMP electrolyte thus represents an optimal balance point where suppression of water reactivity and preservation of ion transport are simultaneously achieved, leading to improved CE and long cycle life. This dual‐scale regulation highlights the importance of jointly considering both the bulk solution environment and the cation coordination structure in AAMB electrolytes.

## 3 Conclusions

In summary, this study presents a dual‐scale electrolyte optimization framework that significantly improves the interfacial stability and electrochemical reversibility of Al electrodes in aqueous Al(OTf)_3_ electrolytes. TMP is identified as an effective cosolvent owing to its favorable miscibility, low viscosity, and intrinsic flame‐retardant properties. From the bulk solution perspective, TMP disrupts the HB network of water and lowers water activity, thereby mitigating HER and corrosion while maintaining sufficiently high ionic conductivity. From the cation solvation perspective, TMP partially replaces coordinated water in the primary solvation structure of Al^3+^, reducing the amount of highly reducible solvated water and suppressing parasitic interfacial reactions. The concurrent regulation of bulk solution chemistry and cation solvation behavior promotes the formation of a robust hybrid organic–inorganic SEI, which stabilizes Al stripping and plating. Among the investigated formulations, 40TMP achieves the most balanced combination of suppressed water reactivity, moderate desolvation penalty, and fast ion transport, enabling symmetric cell cycling exceeding 500 h. Full‐cell evaluations further confirm the advantages of this balanced electrolyte design. When paired with a PANI cathode, the 40TMP electrolyte supports higher CE and significantly improved cycling stability compared with BE. Additional enhancement is achieved by introducing an Sn interfacial layer on the Al anode, which provides favorable nucleation sites and alleviates surface passivation, enabling the Sn@Al|40TMP|PANI cell to retain 70% of its capacity after 400 cycles. Overall, this work highlights the importance of explicitly separating and concurrently regulating bulk solution effects and cation solvation dynamics and provides practical guidance for the rational design of AAMB electrolytes.

## Author Contributions

**Bei‐Er Jia**: conceptualization, methodology, data curation, investigation, validation, visualization, writing – original draft, writing – review and editing, formal analysis. **Dongping Chen**: methodology, investigation. **Gang Wu**: visualization, writing – review and editing, formal analysis, data curation, software, methodology, investigation, validation. **Jin Jie Liew**: investigation, methodology. **Anchun Tang**: investigation. **Huoliang Gu**: investigation, methodology, data curation. **Yue Hu**: data curation, investigation. **Erhai Hu**: investigation. **Jinpeng Song**: investigation. **Chade Lv**: writing – review and editing. **Dan‐Yang Wang**: investigation. **Zhenxiang Xing**: methodology, data curation, investigation, visualization. **Jinxuan Song**: investigation, methodology. **Hong Han Choo**: investigation. **Qiang Zhu**: resources. **Man‐Fai Ng**: methodology, software, data curation, investigation, validation, visualization, resources, writing – review and editing. **Qingyu Yan**: conceptualization, supervision, funding acquisition, project administration, resources, writing – review and editing. **Yuzhu Liu**: investigation. **Chunshuang Yan**: writing – review and editing, resources.

## Funding

A*STAR MTC Programmatic Project (No. M23L9b0052), the Indonesia–NTU Singapore Institute of Research for Sustainability and Innovation (INSPIRASI) (No. 6635/E3/KL.02.02/2023), the Singapore NRF Singapore–China Flagship Program (No. 023740‐00001), and the Ministry of Education (MOE), Singapore, under the Academic Research Fund (AcRF) Tier 2 (MOE‐T2EP50223‐0003).

## Conflicts of Interest

The authors declare no conflicts of interest.

## Supporting information




**Supporting File**: advs76540‐sup‐0001‐SuppMat.pdf.

## Data Availability

The data that support the findings of this study are available from the corresponding author upon reasonable request.
