# Supplementary material for: Balancing Bulk Solution Chemistry and Cation Solvation for Stable Aqueous Aluminum Batteries
Source: Adv Sci (Weinh). 2026 Jul 9:e76540. Online ahead of print. doi: 10.1002/advs.76540 (PMC13348345; doi:10.1002/advs.76540)
Supplement: Supplementary file 1 — Supporting File: advs76540‐sup‐0001‐SuppMat.pdf. [file ADVS-9999-e76540-s001.pdf]

Supporting Information

Balancing Bulk Solution Chemistry and Cation Solvation for Stable Aqueous Aluminum Batteries

Bei-Er Jia†, Gang Wu†, Huoliang Gu, Jin Jie Liew, Dongping Chen, Jinxuan Song, Dan-Yang Wang, Erhai Hu, Hong Han Choo, Yuzhu Liu, Yue Hu, Jinpeng Song, Anchun Tang, Zhenxiang Xing, Qiang Zhu, Chunshuang Yan, Chade Lv, Man-Fai Ng, and Qingyu Yan**

Sn@Al preparation: Sn@Al was prepared via a chemical displacement reaction. First, 2.34 mL of tin(IV) chloride ($SnCl_4$, 98%, Sigma-Aldrich) was added to 200 mL of deionized water to prepare a 0.1 M $SnCl_4$ solution. An Al foil (100 µm, Qinghe Hao Xuan Metal Materials) was polished using sandpaper and cleaned with ethanol. One side of the foil was covered with Kapton tape. The foil was then immersed in the $SnCl_4$ solution for 45 minutes, during which a tin interphase was formed on the exposed side.

PANI preparation: 0.73 mL of aniline (≥99.5%, Sigma-Aldrich) was added to 30 mL of 1 M hydrochloric acid (HCl, Sigma-Aldrich) solution under stirring and cooled in an ice bath. After stirring for 30 min, 10 mL of 1 M HCl containing 0.456 g of ammonium persulfate ($(NH_4)_2S_2O_8$, Sigma-Aldrich) was added dropwise to the above solution. After reacting for 1 hour, the resulting precipitate was collected by centrifugation, washed thoroughly with deionized water and ethanol, and then dried under vacuum at 60 °C for 12 hours.

Electrolyte preparation: Aluminium trifluoromethanesulfonate ($Al(OTf)_3$, 99%) was purchased from Thermo Scientific. Trimethyl phosphate (TMP, ≥99%), triethyl phosphate (TEP, ≥99.8%), tripropyl phosphate (TPP, 99%), and tributyl phosphate (TBP, ≥99%) were obtained from Sigma-Aldrich. To prepare the electrolytes, 1 M $Al(OTf)_3$ was dissolved in a mixed solvent of x vol% TMP and $(100 - x)$ vol% deionized water (denoted as xTMP, where x = 20, 40, 60, or 80). Specifically, 0.474 g of $Al(OTf)_3$ powder was added to $10x$ µL of TMP and $(1000 - 10x)$ µL of deionized water. Electrolytes containing TEP, TPP, or TBP were

prepared following the same nomenclature and procedure. A 1 M $Al(OTf)_3$ aqueous solution without any cosolvent was used as the baseline electrolyte (denoted as BE).

Characterization: Fourier transform infrared (FTIR) spectroscopy was performed on a PerkinElmer Frontier FTIR spectrometer. Raman spectra were recorded using a confocal Raman microscope (WITec Alpha300 SR) with a 633 nm excitation laser. The ^{1}H and ^{27}Al nuclear magnetic resonance (NMR) spectra were obtained on a JEOL JNM-ECA500II spectrometer. For each measurement, 500 μL of electrolyte was transferred into a standard 5 mm NMR tube. Deuterium oxide (99.9 atom% D, Sigma-Aldrich) contained in a coaxial insert was employed as an external field-frequency lock. Differential scanning calorimetry (DSC, TA Instruments DSC Q10) was used to determine the freezing point of the electrolyte over a temperature range from −80 °C to 40 °C, using a heating rate of 5 °C min^{-1} under a nitrogen atmosphere. The contact angle was measured using a DataPhysics OCA 15Pro goniometer. Hydrogen evolution was monitored using differential electrochemical mass spectrometry (DEMS) with a PM-DEMS system (Shanghai Pro-tech Limited Company). Measurements were conducted on Al||Al symmetric cells subjected to galvanostatic cycling at a current density of 0.05 mA cm^{-2}, following a repeated protocol of 0.5 h discharge and 0.5 h charge steps. The ionic conductivity of the electrolytes was measured using a portable conductivity meter (INESA DDBJ-350F). X-ray diffraction (XRD) patterns were acquired using an X-ray diffractometer (Bruker D2 PHASER) equipped with a Cu Kα radiation source (λ=1.5406 Å). Scanning electron microscopy (SEM) and energy-dispersive X-ray spectroscopy (EDS) analyses were performed using a field-emission SEM (JEOL JSM-7600F). X-ray photoelectron spectroscopy (XPS) spectra were collected using an XPS spectrometer (Kratos Axis Supra) equipped with an Al Kα X-ray source. Depth profiling was conducted with Ar^{+} sputtering (5 keV), employing a raster size of 2 mm. XPS spectra were charge corrected by referencing to the adventitious C 1s (C–C) peak at 284.8 eV. Time-of-flight secondary ion mass spectrometry (ToF-SIMS, IONTOF TOF.SIMS 5) was further employed to investigate the depth distribution of the interphase. Negative ion depth profiling was conducted with a 2 keV Cs^{+} sputtering ion beam, rastered over an area of 300 μm × 300 μm. A pulsed 30 keV Bi_1^{+} primary ion beam was employed for analysis, scanned over a 100 μm × 100 μm area.

Electrochemical measurements: The PANI cathode was prepared by mixing PANI, Super P (>99%, Alfa Aesar), and polyvinylidene fluoride (PVDF, Tianjin EVS Chemical Technology) in a mass ratio of 7:2:1 with an appropriate amount of 1-Methyl-2-pyrrolidinone (NMP, >99%,

Sigma-Aldrich). The resulting slurry was uniformly coated onto a titanium foil (10 μm, Qinghe Hao Xuan Metal Materials) and dried in a vacuum oven at 80 °C overnight. The loading mass of the active material was ~0.8 mg cm^{-2}. 2032-type coin cells were assembled using Whatman GF/A glass microfiber filters as separators. The electrolyte volume was fixed at 50 μL. Prior to use, the bare Al foils (100 μm, Qinghe Hao Xuan Metal Materials) were polished. In symmetric cells, Al foil was used for both the anode and cathode. Galvanostatic charge/discharge (GCD) measurements were performed using a Neware BTS4000 battery testing system. The full cell tests were conducted within a voltage window of 0.1–1.1 V. Other electrochemical tests, including cyclic voltammetry (CV), linear sweep voltammetry (LSV), electrochemical impedance spectroscopy (EIS), and linear polarization, were carried out on an Autolab PGSTAT204 workstation. CV was performed at a scan rate of 0.2 mV s^{-1}. Tafel plots were obtained from linear polarization tests on Al||Al cells (8 mm Al discs) at 0.5 mV s^{-1}. LSV was conducted on Ti||Al cells at 5 mV s^{-1} with an 8 mm Al disc working electrode and a 12 mm Ti disc counter/reference electrode. All electrochemical tests were conducted at room temperature (25 °C).

Density functional theory (DFT) calculations: Geometry optimizations are performed using the unrestricted B3LYP hybrid density functional[1, 2] implemented in Gaussian 16 suite of program. No symmetry constrain is applied. The 6−311+G (d,p) basis sets are adopted for all atoms.[3, 4] "Tight" optimizations and "ultrafine" integration grid are specified for the DFT calculations. GD3 empirical dispersion corrections are applied.[5] The implicit universal water solvation model based on SMD is applied.[6] The method is applied to calculate the LUMO and the total energy of the molecule/ion. The binding energy (E_b) is calculated using the equation: $E_b = E_{AB} - (E_A + E_B)$ where E_{AB} and $E_A(E_B)$ are the total energies of the binded molecule/ion AB and the isolated molecule/ion A(B), respectively. The more negative the binding energy, the stronger the binding strength.

Molecular dynamics (MD) simulations: All molecular dynamics simulations were performed using the LAMMPS (Large-scale Atomic/Molecular Massively Parallel Simulator) software package.[7, 8] Initial configurations of electrolyte mixtures were generated using PackMol[9] to achieve homogeneous spatial distributions. The COMPASS (Condensed-phase Optimized Molecular Potentials for Atomistic Simulation Studies)[10] force field was employed to describe interatomic interactions. Atomic partial charges were assigned based on RESP2$_{0.5}$ charge (B3LYP-D3(BJ)/def2-TZVP, SMD-water). Periodic boundary conditions were applied

in all three dimensions, and long-range electrostatic interactions were treated using the particle–particle particle–mesh (PPPM) method. Simulations were conducted in the NPT ensemble at 298 K and 1 atm, with temperature and pressure controlled via the Nose–Hoover thermostat and barostat.[11, 12] The integration timestep was set to 1.0 fs. Four configurations were generated for each electrolyte formula to ensure a reliable ensemble average. Each system was initialized at a low packing density (0.6 g cm^{-3}) and gradually relaxed to a higher equilibrium density through sequential NPT equilibration for 40 ns.

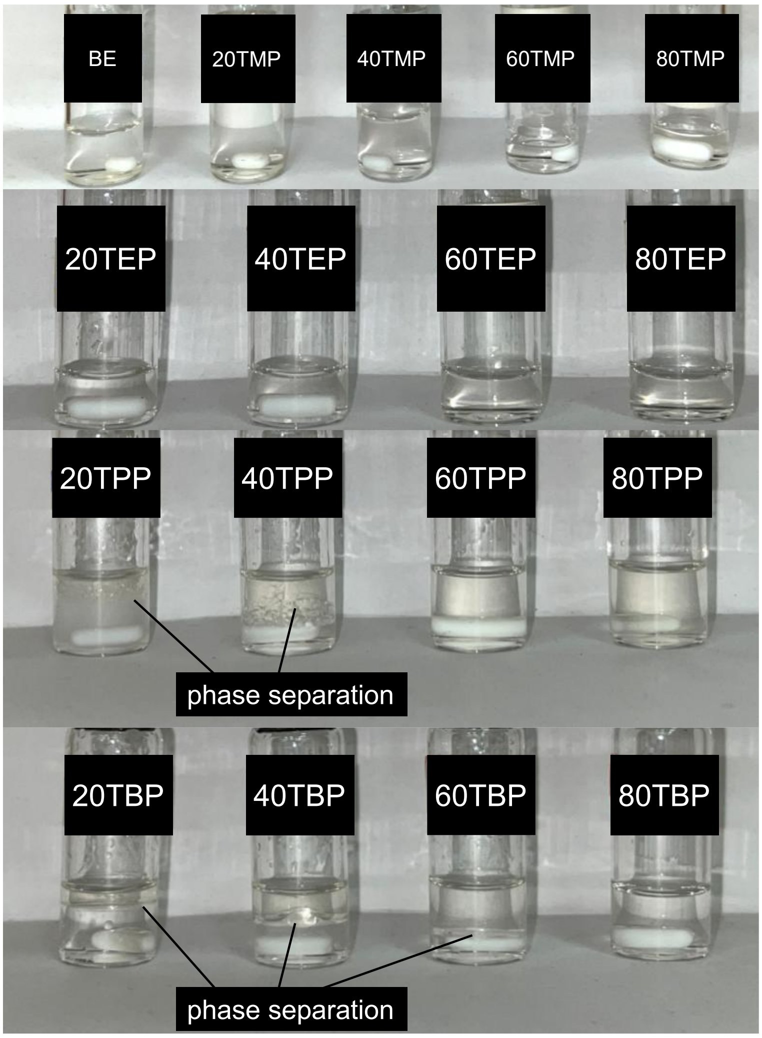

Figure S1. Photographs of the prepared electrolytes.

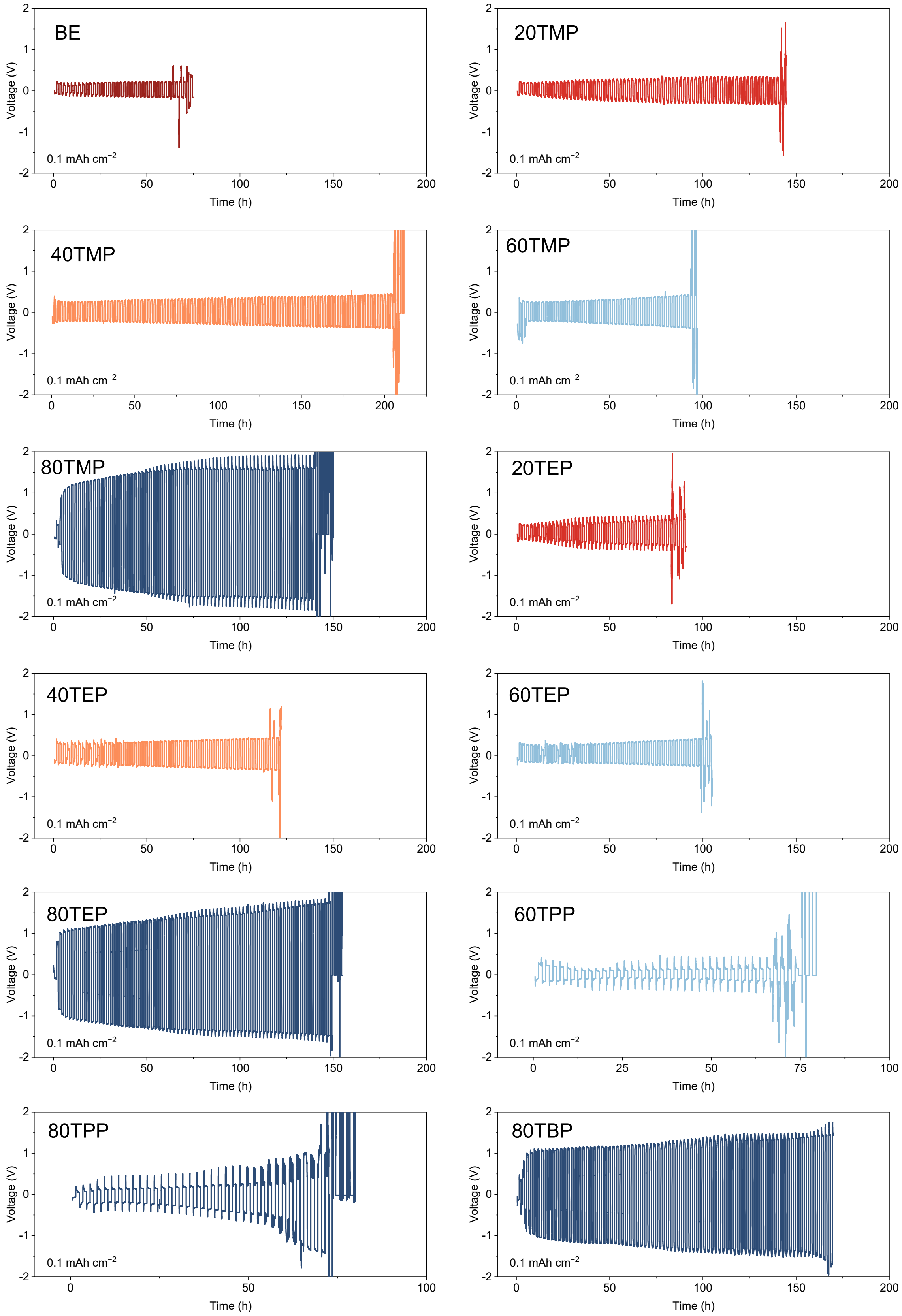

Figure S2. Voltage profiles of Al||Al symmetric cells with 12 electrolytes, cycled at a current density of 0.1 mA cm^{-2} and an areal capacity of 0.1 mAh cm^{-2}.

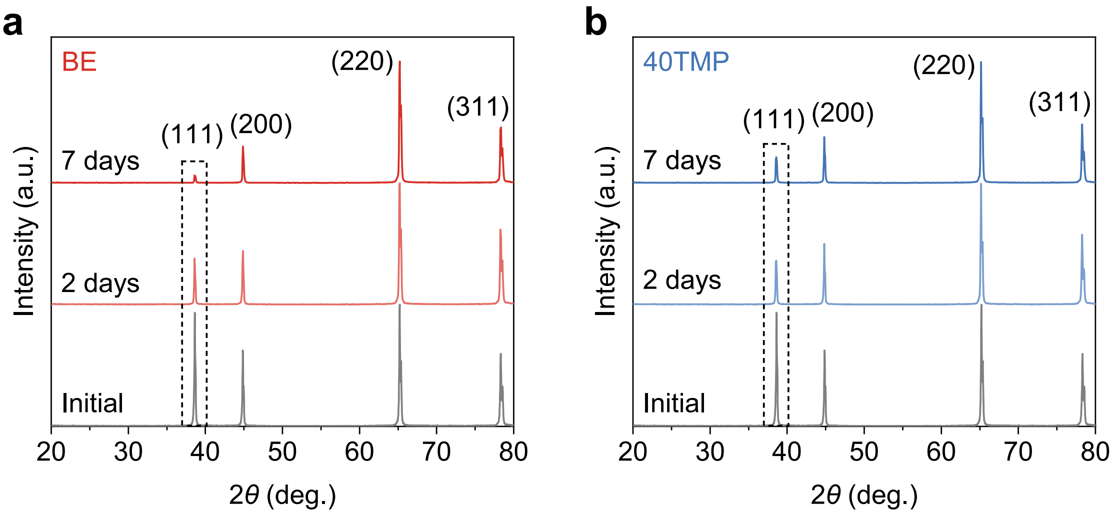

Figure S3. XRD patterns of Al electrodes after soaking in (a) BE and (b) 40TMP electrolytes for 0, 2, and 7 days, showing the evolution of diffraction peaks with soaking time.

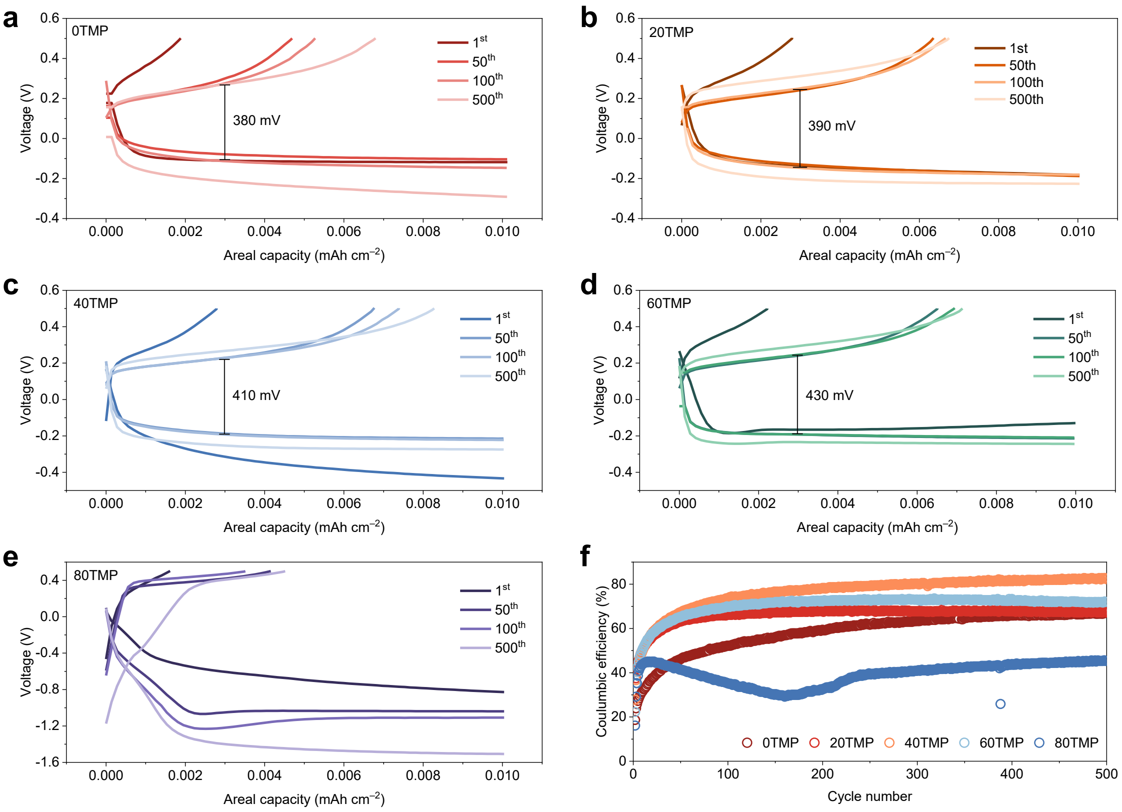

Figure S4. (a–e) Voltage profiles of Al plating/stripping in Al||Cu asymmetric cells with different TMP contents. (f) Coulombic efficiency of Al plating/stripping in Al||Cu asymmetric cells with different TMP contents.

Among the tested electrolytes, 40TMP shows the highest average Coulombic efficiency, indicating the most favorable balance between parasitic reaction suppression and interfacial plating/stripping kinetics.

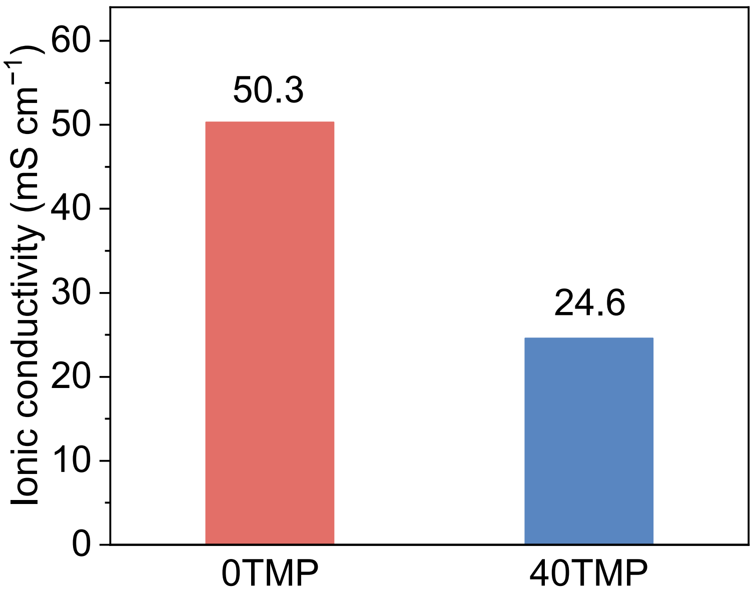

Figure S5. Ionic conductivity of the electrolytes measured at 25 °C.

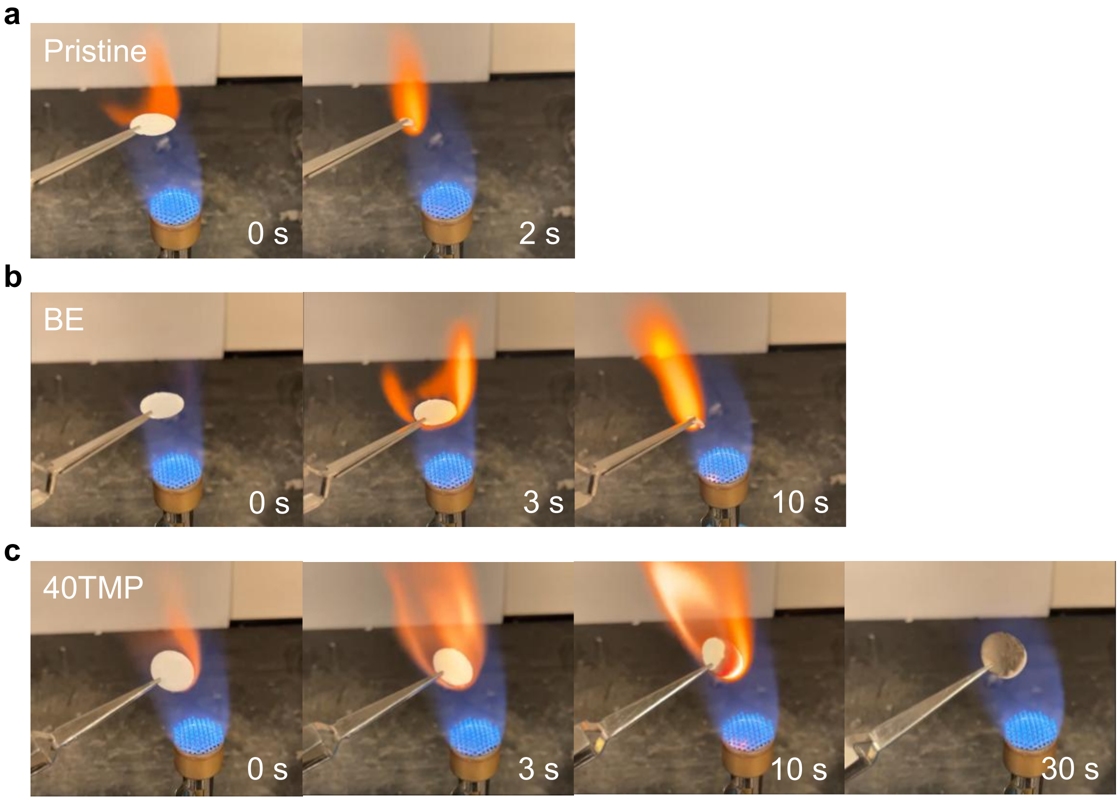

Figure S6. Combustion tests of (a) the pristine separator, (b) the separator with BE electrolyte, and (c) the separator with 40TMP electrolyte, performed using a Bunsen burner.

The pristine and BE-treated separators burned rapidly and were completely consumed by the flame. In contrast, the 40TMP-treated separator exhibited excellent flame retardancy, remaining structurally intact after 30 seconds of continuous burning, with only surface darkening observed. The improved flame resistance of the 40TMP-treated separator is attributed to the presence of TMP, a well-known flame retardant.

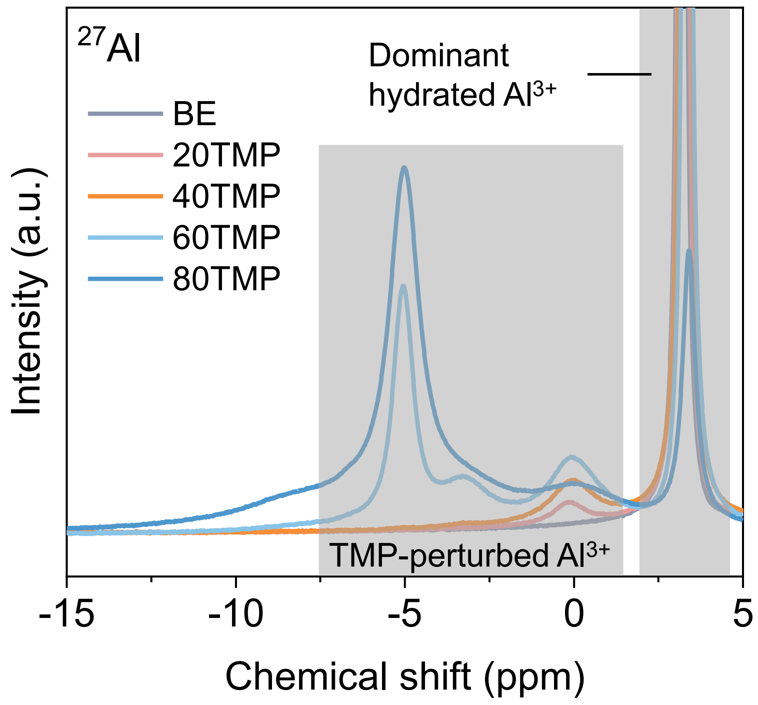

Figure S7. ^{27}Al NMR spectra of electrolytes with varying TMP contents.

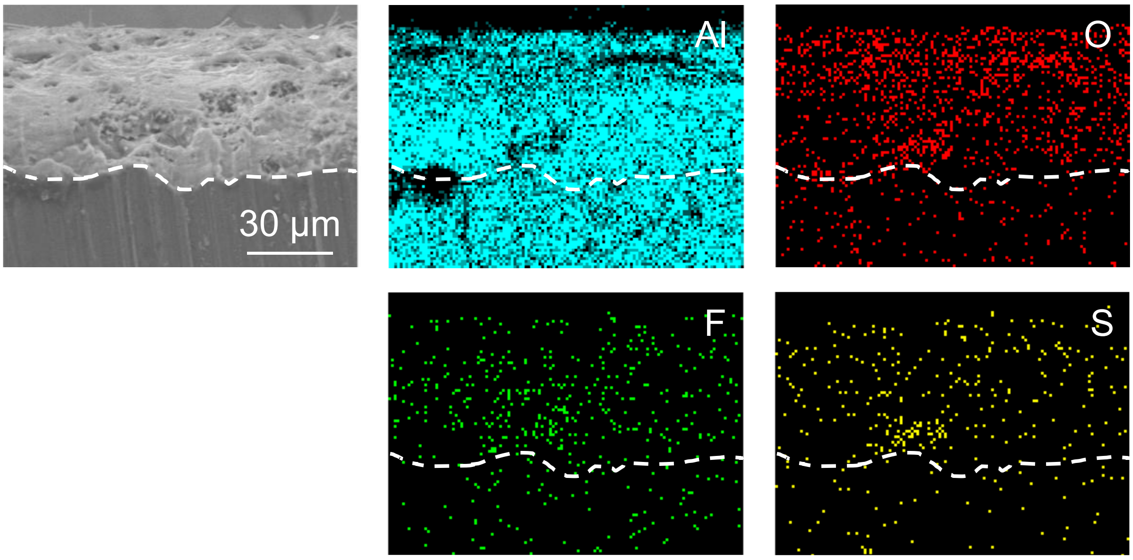

Figure S8. Cross-sectional SEM image and corresponding EDS elemental maps of the Al electrode after cycling in an Al|BE|Al symmetric cell (0.05 mA cm^{-2}, 0.1 mAh cm^{-2}, 25 cycles).

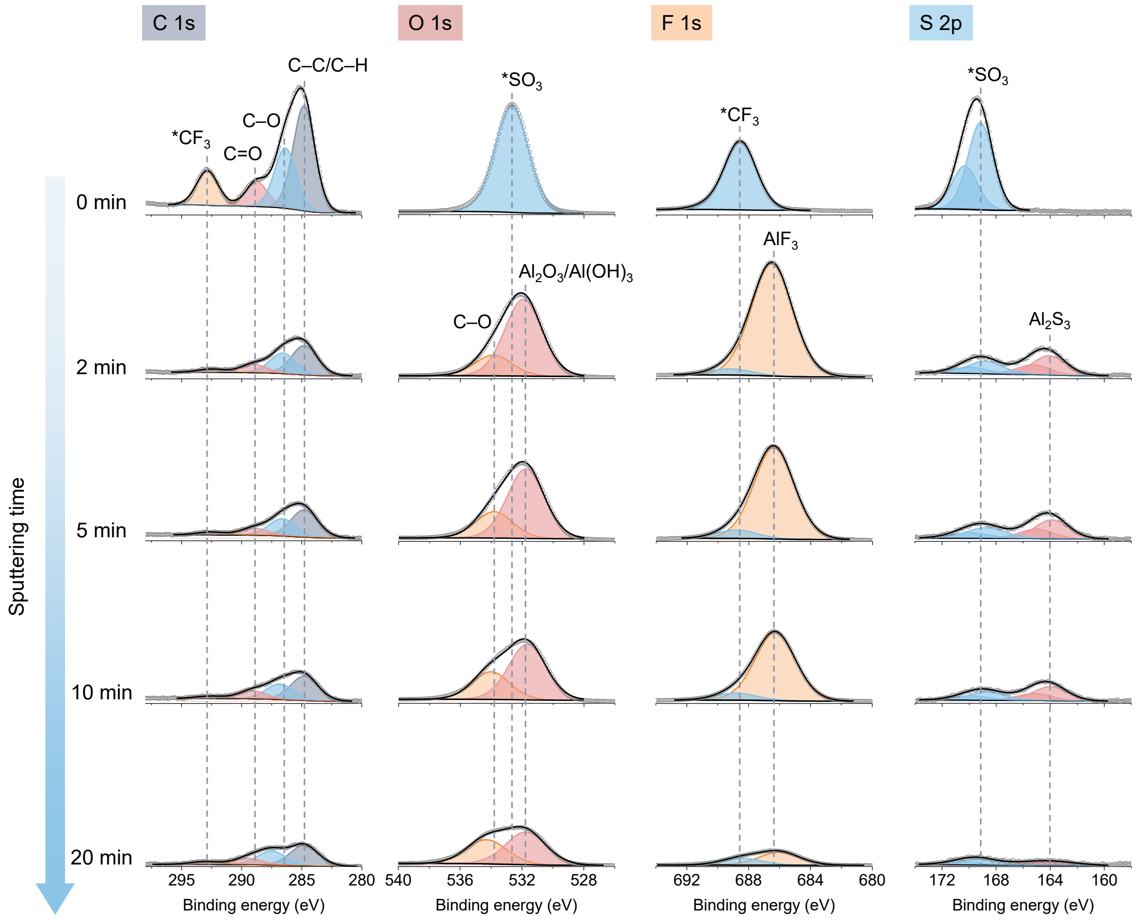

Figure S9. High-resolution C 1s, O 1s, F 1s, and S 2p XPS spectra of the Al electrode cycled in BE, collected after 0, 2, 5, 10, and 20 min of Ar^+ sputtering.

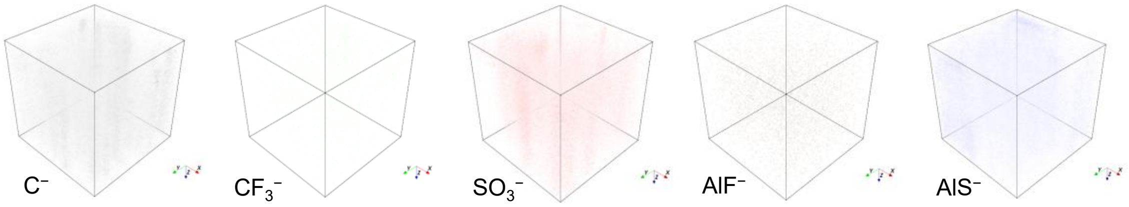

Figure S10. ToF-SIMS 3D elemental distributions of representative SEI fragments on the Al electrode cycled in BE.

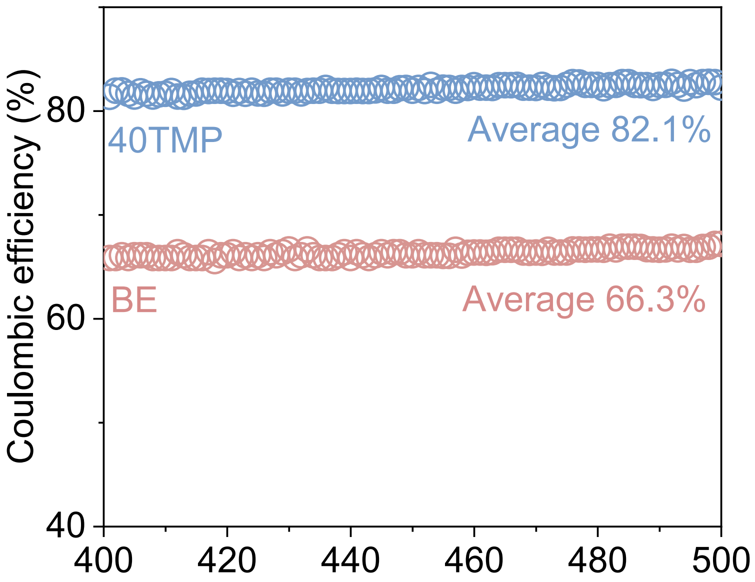

Figure S11. Coulombic efficiency of Al plating/stripping in Al||Cu asymmetric cells using BE and 40TMP electrolytes over cycles 400–500.

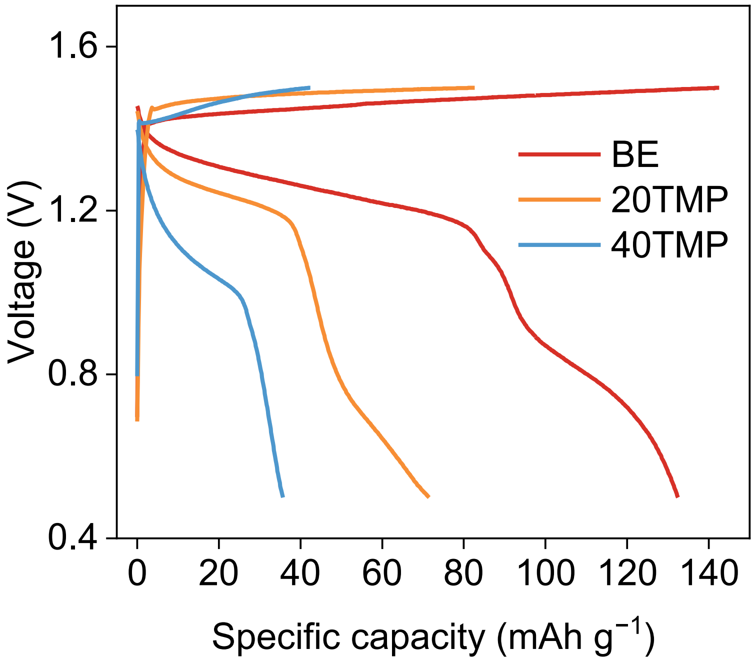

Figure S12. Second-cycle galvanostatic charge/discharge profiles of Al||Al_xMnO_2 full cells using BE, 20TMP, and 40TMP electrolytes at a current density of 0.1 A g^{-1}.

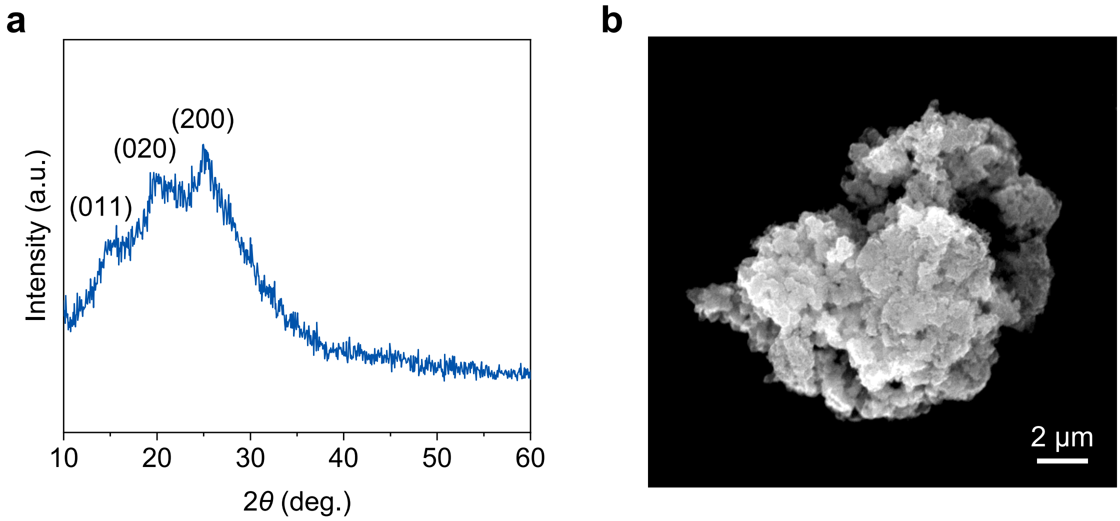

Figure S13. (a) XRD pattern of the synthesized PANI. It exhibits a characteristic broad diffraction peak at around 25°, which is attributed to the π–π stacking interactions between polymer chains. (b) SEM image of the synthesized PANI.

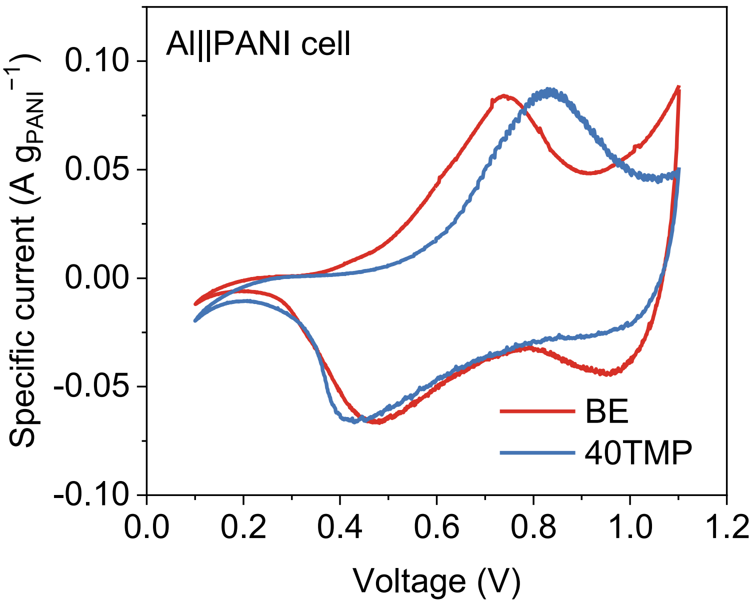

Figure S14. CV profiles of the Al||PANI full cell at 0.2 mV s^{-1}.

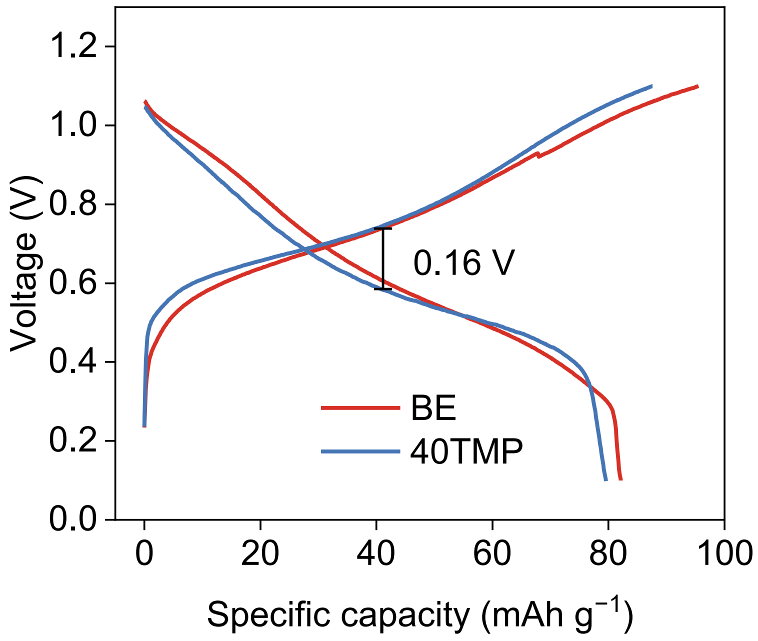

Figure S15. Representative voltage profiles of Al||PANI cells at a specific current of 0.1 A g^{-1}.

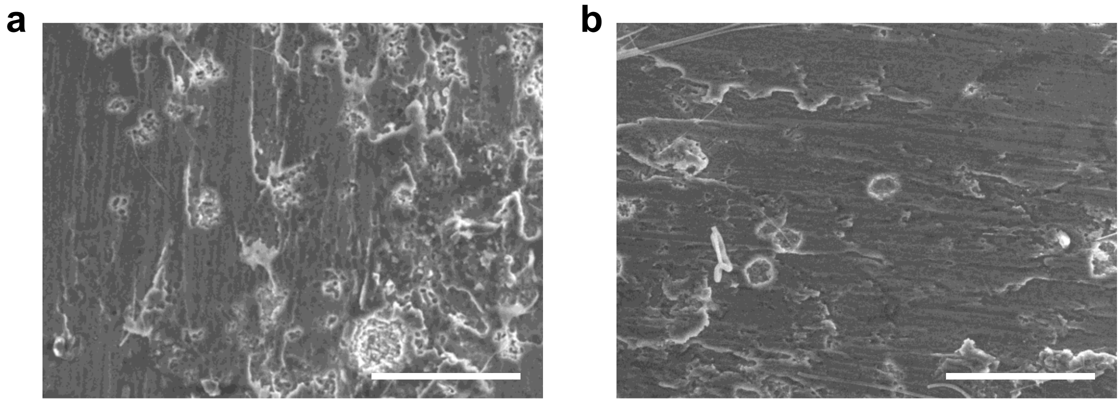

Figure S16. Top-view SEM images of Al electrodes from full cells using (a) BE and (b) 40TMP electrolytes. Scale bars: 50 μm.

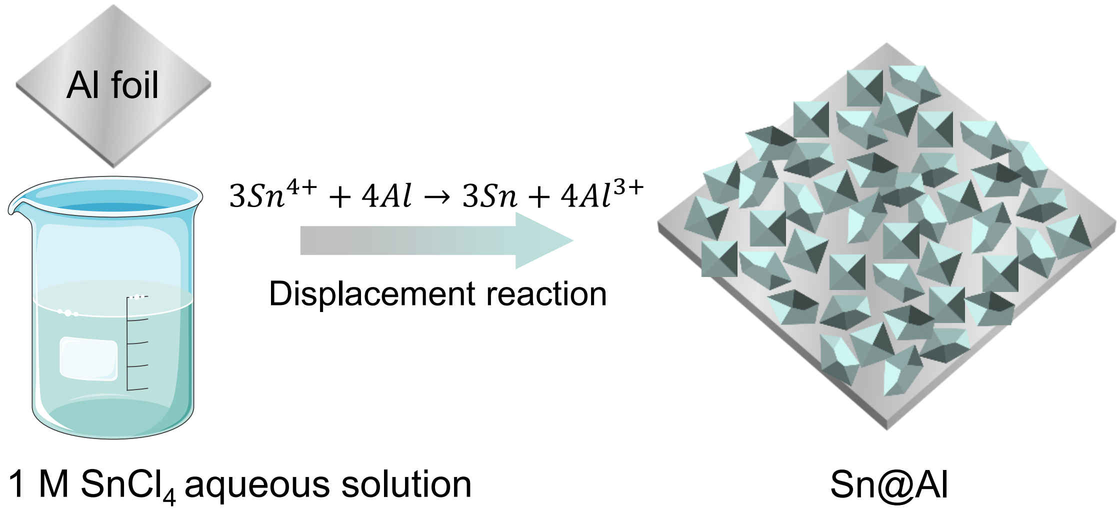

Figure S17. Schematic illustration of the displacement reaction used to synthesize Sn@Al by immersing Al in $SnCl_4$ aqueous solution.

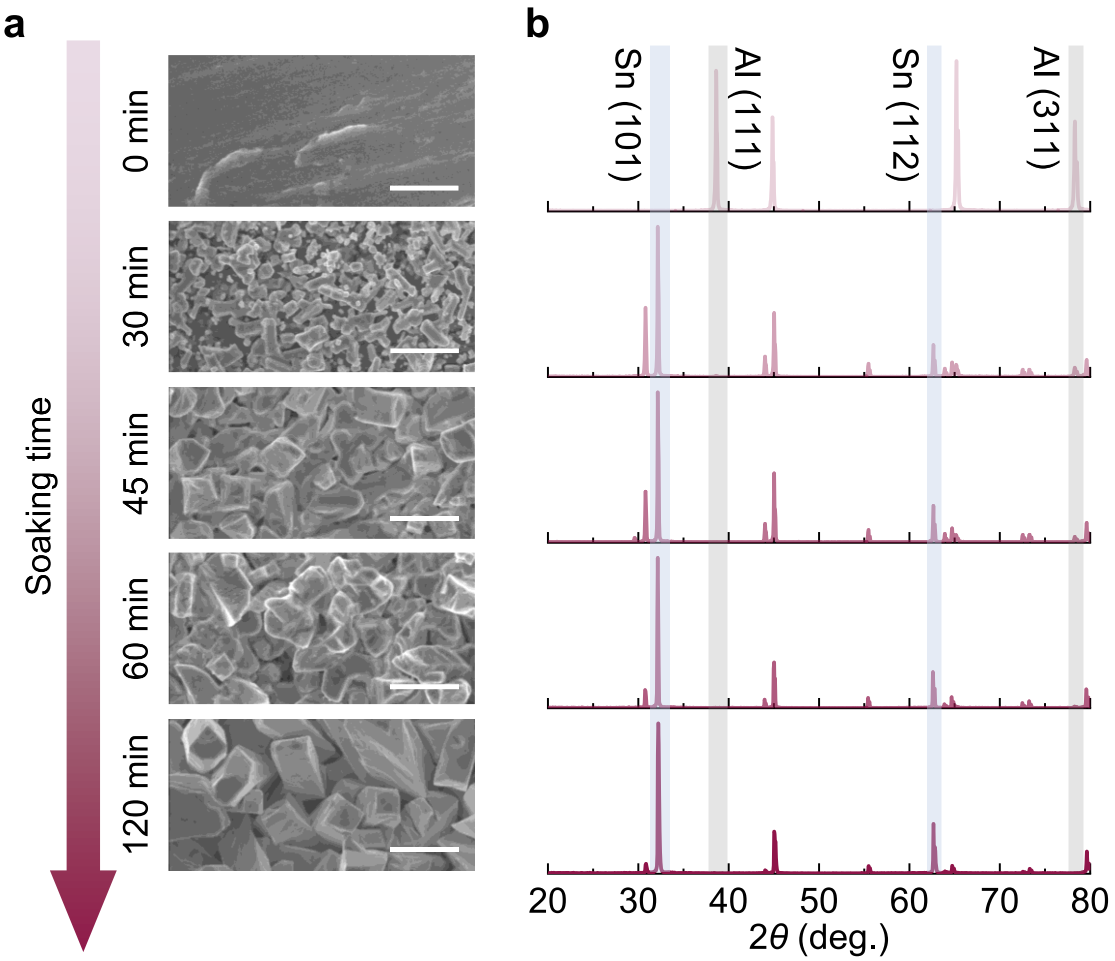

Figure S18. (a) SEM images and (b) XRD patterns of Sn@Al samples obtained with increasing soaking time. Scale bars: 10 μm.

With increasing soaking time, the Sn coverage on Al progressively increases, accompanied by the growth of Sn grain size. Characteristic diffraction peaks of Sn emerge and intensify, while those of Al weaken and eventually disappear.

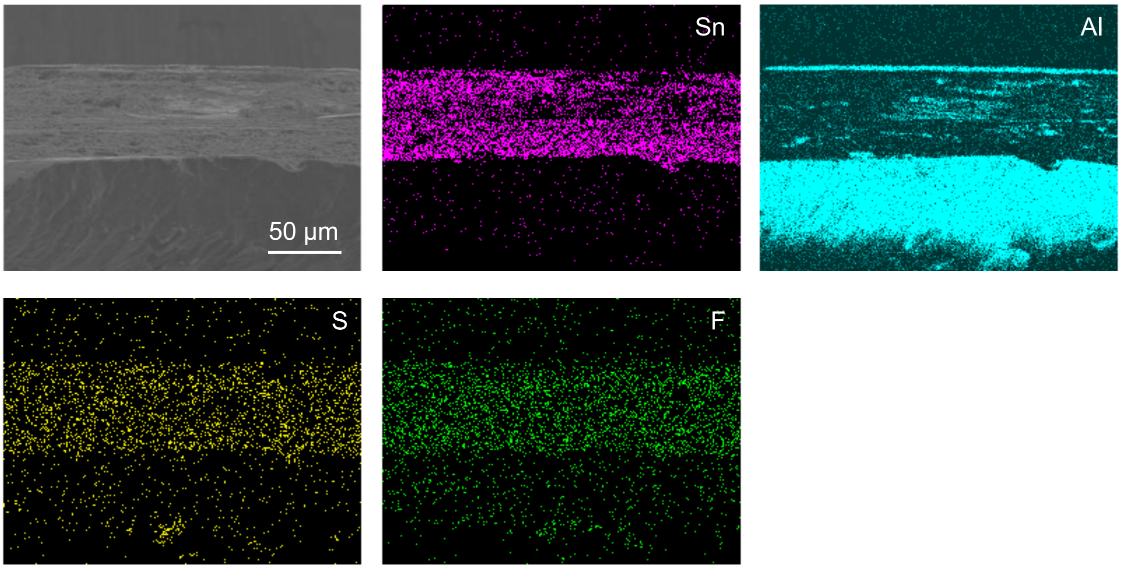

Figure S19. Cross-sectional SEM image and corresponding EDS elemental mappings of Sn, Al, S, and F for the Sn@Al anode recovered from the Sn@Al|40TMP|PANI full cell after cycling.

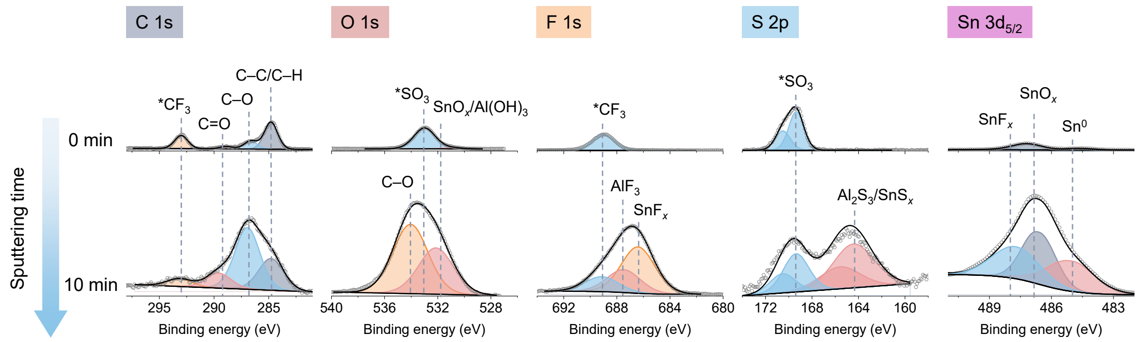

Figure S20. XPS spectra of the cycled Sn@Al electrode before and after Ar^+ sputtering, including C 1s, O 1s, F 1s, S 2p, and Sn $3d_{5/2}$ regions.

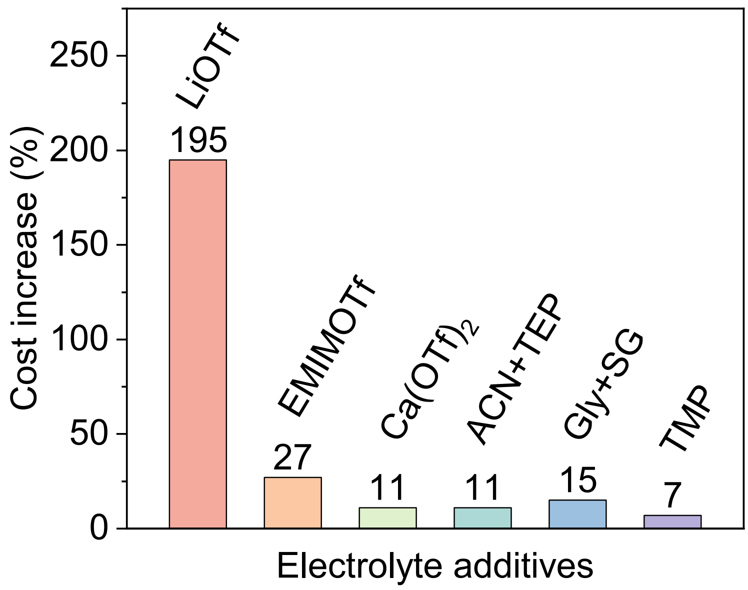

Figure S21. Relative cost increase of TMP and other reported additives, each referenced to the baseline electrolyte used in its corresponding literature.

Table S1. Summary of physicochemical properties for common cosolvents reported in aqueous battery electrolytes.

Cosolvent	Melting Point (°C)	Boiling Point (°C)	Flash Point (°C)	Viscosity (mPa·s, 25 °C)	Donor Number (kcal mol^{-1})	Dielectric Constant (25 °C)	Non-flammable	Water miscibility
1,2-Dimethoxyethane (DME)	−58	85	−2	0.45	20	7.2	✕	✓
1,4-Dioxane	11.8	101	12	1.20	14.8	2.2	✕	✓
Acetone	−95	56	−20	0.31	17	21.4	✕	✓
Acetonitrile (ACN)	−45	82	2	0.37	14.1	38.8	✕	✓
Butanone (MEK)	−86	80	−9	0.43	17.4	18.5	✕	✕
Cyclohexane	6.5	80.7	−20	0.98	0	1.9	✕	✕
Diethyl carbonate (DEC)	−43	126	25	0.75	16	3.1	✕	✕
Diethyl ether (DEE)	−116.3	34.6	−45	0.24	19.2	4.3	✕	✕
Dimethyl carbonate (DMC)	2	90	17	0.60	17.2	3.2	✕	✕
Dimethyl sulfoxide (DMSO)	19	189	89	1.99	29.8	46.7	✕	✓
Ethanol	−114	78	14	1.07	19.2	24.5	✕	✓
Ethyl acetate (EA)	−83.6	77	−4	0.43	17.1	6.4	✕	✕
Ethylene carbonate (EC)	36.4	/	143	1.90	16.4	89.8	✓	✕
Ethylene glycol (EG)	−13	197	111	16.1	19.2	37	✕	✓
Formamide (FA)	2.6	210	154	3.3	24	111	✓	✓
Glycerol (Gly)	17.8	290	160	1412	19	47	✓	✓
Methanol	−97.6	64.7	11	0.55	19	32.6	✕	✓
N,N-Dimethylformamide (DMF)	−61	153	58	0.92	26.6	36.7	✕	✓
N-Methyl-2-pyrrolidone (NMP)	−24	202	91	1.67	27.3	32.2	✕	✓
Propylene carbonate (PC)	−49	242	132	2.5	15.1	64.9	✓	✓
Tetrahydrofuran (THF)	−108.4	66	−14	0.48	20	7.6	✕	✓
Triethyl phosphate (TEP)	−56.5	215	107	1.77	26	13.0	✓	✓
Trimethyl phosphate (TMP)	−46	197	115	2.16	23	21.3	✓	✓
Water	0	100	/	0.89	18	78.4	✓	✓

Table S2. Comparison of the ionic conductivity of aqueous $Al(OTf)_3$-based electrolytes with different additives.

Composition	Ionic conductivity at 25°C (mS cm^{-1})	References
1 M $Al(OTf)_3$ + 15 M LiOTf	2	[13]
$Al(OTf)_3$ in (acetonitrile+H_2O)	6.6	[14]
$Al(OTf)_3$: Acetonitrile: Triethyl phosphate: H_2O=1:4:4:40	9.61	[15]
($Al(OTf)_3$: Glycerol: β-Glycerol phosphate disodium salt pentahydrate=1:8:1) + 30 vol% H_2O	6.34	[16]
1 M $Al(OTf)_3$ in (40 vol% Trimethyl phosphate+60 vol% H_2O)	24.6	This work

Table S3. Numbers of ions and molecules employed in the MD simulation models. The close agreement between the simulated and experimental densities confirms the reasonableness and reliability of the simulation.

Electrolyte	Experimental density (g cm^{-3})	Simulated density (g cm^{-3})	Al^{3+}	OTf^{-}	H_2O	TMP
BE	1.22	1.19	18	54	1000	/
20TMP	1.25	1.23	23	69	1000	39
40TMP	1.29	1.27	30	90	500	103
60TMP	1.32	1.31	23	69	500	116
80TMP	1.37	1.35	30	90	333	206

Table S4. Al^{3+}–ligand coordination numbers in electrolytes averaged over four configurations.

Electrolyte	Configuration	Al^{3+}–O (H_2O)	Al^{3+}–O (OTf^-)	Al^{3+}–O (TMP)
BE	Config. 1	5.42	1.06	/
	Config. 2	5.23	1.30	/
	Config. 3	5.35	1.15	/
	Config. 4	5.43	1.05	/
	Average	5.36	1.14	/
20TMP	Config. 1	5.20	1.06	0.17
	Config. 2	5.20	1.06	0.17
	Config. 3	5.14	1.12	0.18
	Config. 4	5.09	1.18	0.18
	Average	5.16	1.10	0.18
40TMP	Config. 1	4.82	1.09	0.47
	Config. 2	4.76	1.18	0.45
	Config. 3	4.78	1.15	0.46
	Config. 4	4.82	1.10	0.46
	Average	4.80	1.13	0.46
60TMP	Config. 1	4.00	1.23	1.07
	Config. 2	4.04	1.25	1.02
	Config. 3	4.00	1.33	1.00
	Config. 4	4.01	1.31	1.00
	Average	4.01	1.28	1.02
80TMP	Config. 1	2.52	1.36	2.32
	Config. 2	2.58	1.25	2.35
	Config. 3	2.56	1.24	2.38
	Config. 4	2.48	1.25	2.43
	Average	2.54	1.28	2.37

Table S5. Additive-induced cost increase of electrolytes, based on reported aqueous aluminum battery formulations. Pricing information sourced from the Sigma-Aldrich website.

Aluminum salt	Cost (SGD)	Additive	Cost (SGD)	Cost increase	Reference
$AlCl_3$	92.3/100 g	Lithium bis(trifluoromethanesulfonyl)imide (LiTFSI)	698/100 g	39000%	[17]
$Al(OTf)_3$	800/50 g	Lithium trifluoromethanesulfonate (LiOTf)	631/100 g	195%	[13]
		1-Ethyl-3-methylimidazolium trifluoromethanesulfonate (EMIMOTf)	433/50 g	27%	[18]
		Calcium trifluoromethanesulfonate ($Ca(OTf)_2$)	494/50 g	11%	[19]
		Acetonitrile (ACN)<br>+<br>Triethyl phosphate (TEP)	269/1 L<br>115/100 mL	11%	[15]
		Glycerol (Gly)<br>+<br>β-Glycerol phosphate disodium salt pentahydrate (SG)	301/500 mL<br>258/100 g	15%	[16]
		Trimethyl phosphate (TMP)	57.5/50 g	7%	This work

References

[1] A.D. Becke, Density-functional thermochemistry. III. The role of exact exchange, J. Chem. Phys., 98 (1993) 5648-5652. 10.1063/1.464913

[2] P.J. Stephens, F.J. Devlin, C.F. Chabalowski, M.J. Frisch, Ab Initio Calculation of Vibrational Absorption and Circular Dichroism Spectra Using Density Functional Force Fields, J. Phys. Chem., 98 (1994) 11623-11627. 10.1021/j100096a001

[3] A.D. McLean, G.S. Chandler, Contracted Gaussian basis sets for molecular calculations. I. Second row atoms, Z=11–18, J. Chem. Phys., 72 (1980) 5639-5648. 10.1063/1.438980

[4] R. Krishnan, J.S. Binkley, R. Seeger, J.A. Pople, Self-consistent molecular orbital methods. XX. A basis set for correlated wave functions, J. Chem. Phys., 72 (1980) 650-654. 10.1063/1.438955

[5] S. Grimme, J. Antony, S. Ehrlich, H. Krieg, A consistent and accurate ab initio parametrization of density functional dispersion correction (DFT-D) for the 94 elements H-Pu, J. Chem. Phys., 132 (2010). 10.1063/1.3382344

[6] A.V. Marenich, C.J. Cramer, D.G. Truhlar, Universal Solvation Model Based on Solute Electron Density and on a Continuum Model of the Solvent Defined by the Bulk Dielectric Constant and Atomic Surface Tensions, J. Phys. Chem. B, 113 (2009) 6378-6396. 10.1021/jp810292n

[7] S. Plimpton, Fast Parallel Algorithms for Short-Range Molecular Dynamics, J. Comput. Phys., 117 (1995) 1-19. https://doi.org/10.1006/jcph.1995.1039

[8] A.P. Thompson, H.M. Aktulga, R. Berger, D.S. Bolintineanu, W.M. Brown, P.S. Crozier, P.J. in 't Veld, A. Kohlmeyer, S.G. Moore, T.D. Nguyen, R. Shan, M.J. Stevens, J. Tranchida, C. Trott, S.J. Plimpton, LAMMPS - a flexible simulation tool for particle-based materials modeling at the atomic, meso, and continuum scales, Comput. Phys. Commun., 271 (2022) 108171. https://doi.org/10.1016/j.cpc.2021.108171

[9] L. Martínez, R. Andrade, E.G. Birgin, J.M. Martínez, PACKMOL: A package for building initial configurations for molecular dynamics simulations, J. Comput. Chem., 30 (2009) 2157-2164. https://doi.org/10.1002/jcc.21224

[10] H. Sun, COMPASS: An ab Initio Force-Field Optimized for Condensed-Phase ApplicationsOverview with Details on Alkane and Benzene Compounds, J. Phys. Chem. B, 102 (1998) 7338-7364. 10.1021/jp980939v

[11] S. Nosé, A unified formulation of the constant temperature molecular dynamics methods, J. Chem. Phys., 81 (1984) 511-519. 10.1063/1.447334

[12] W.G. Hoover, Canonical dynamics: Equilibrium phase-space distributions, Phys. Rev. A, 31 (1985) 1695-1697. 10.1103/PhysRevA.31.1695

[13] Y. Gao, Y. Li, H. Yang, L. Zheng, Y. Bai, C. Wu, Bi-salt electrolyte for aqueous rechargeable aluminum battery, J. Energy Chem., 67 (2022) 613-620. https://doi.org/10.1016/j.jechem.2021.11.003

[14] Q. Dou, N. Yao, W.K. Pang, Y. Park, P. Xiong, X. Han, H.H. Rana, X. Chen, Z.-H. Fu, L. Thomsen, B. Cowie, Y. Kang, Q. Liu, D.H. Min, Y.M. Jung, Z. Guo, Q. Zhang, H.S. Park, Unveiling solvation structure and desolvation dynamics of hybrid electrolytes for ultralong cyclability and facile kinetics of Zn–Al alloy anodes, Energy Environ. Sci., 15 (2022) 4572-4583. 10.1039/D2EE02453E

[15] X. Luo, R. Wang, L. Zhang, Z. Liu, H. Li, J. Mao, S. Zhang, J. Hao, T. Zhou, C. Zhang, Air-Stable and Low-Cost High-Voltage Hydrated Eutectic Electrolyte for High-Performance Aqueous Aluminum-Ion Rechargeable Battery with Wide-Temperature Range, ACS Nano, 18 (2024) 12981-12993. 10.1021/acsnano.4c01276

[16] X. Zhang, R. Wang, Z. Liu, Q. Ma, H. Li, Y. Liu, J. Hao, S. Zhang, J. Mao, C. Zhang, Regulated Hydrated Eutectic Electrolyte Enhancing Interfacial Chemical Stability for Highly Reversible Aqueous Aluminum-Ion Battery with a Wide Temperature Range of −20 to 60 °C, Adv. Energy Mater., 14 (2024) 2400314. https://doi-org.remotexs.ntu.edu.sg/10.1002/aenm.202400314

[17] X. Li, Y. Tang, C. Li, H. Lv, H. Fan, W. Wang, T. Cai, Y. Cui, W. Xing, Z. Yan, C. Zhi, H. Li, Relieving hydrogen evolution and anodic corrosion of aqueous aluminum batteries with hybrid electrolytes, J. Mater. Chem. A, 10 (2022) 4739-4748. 10.1039/D1TA10125K

[18] A. Lahiri, S. Guan, A. Chutia, Modulating Aluminum Solvation with Ionic Liquids for Improved Aqueous-Based Aluminum-Ion Batteries, ACS Appl. Energy Mater., 6 (2023) 11874-11881. 10.1021/acsaem.3c01745

[19] X. Yang, H. Gu, Q. Sun, W. Zhang, Z. Li, Synergistic co-embedding of metal ions and hydrogen protons for high stability double salt aqueous aluminum battery, Energy Storage Mater., 61 (2023) 102917. https://doi.org/10.1016/j.ensm.2023.102917
